# Antibacterial Activity and Prebiotic Properties of Six Types of Lamiaceae Honey

**DOI:** 10.3390/antibiotics13090868

**Published:** 2024-09-10

**Authors:** Filomena Nazzaro, Maria Neve Ombra, Francesca Coppola, Beatrice De Giulio, Antonio d’Acierno, Raffaele Coppola, Florinda Fratianni

**Affiliations:** 1 Institute of Food Science, CNR-ISA, Via Roma 64, 83100 Avellino, Italy; nombra@isa.cnr.it (M.N.O.); francesca.coppola@isa.cnr.it (F.C.); bdegiulio@isa.cnr.it (B.D.G.); coppola@unimol.it (R.C.); fratianni@isa.cnr.it (F.F.); 2 Department of Agriculture, Environment and Food (DiAAA), University of Molise, Via de Sanctis, 86100 Campobasso, Italy

**Keywords:** honey, biofilm, antibacterial, probiotics, prebiotics, hydrophobicity, hemolysis, cytotoxicity

## Abstract

Our work investigated the antimicrobial and prebiotic properties of basil, mint, oregano, rosemary, savory, and thyme honey. The potential antimicrobial action, assessed against the pathogens *Acinetobacter baumannii*, *Escherichia coli*, *Listeria monocytogenes*, *Pseudomonas aeruginosa*, and *Staphylococcus aureus*, evidenced the capacity of the honey to influence the pathogenic hydrophobicity and hemolytic activities. Honey inhibited pathogen biofilms, acting especially on the mature biofilms, with inhibition rates of up to 81.62% (caused by the presence of mint honey on *L. monocytogenes*). *S. aureus* biofilms were the most susceptible to the presence of honey, with inhibition rates up of to 67.38% in the immature form (caused by basil honey) and up to 80.32% in the mature form (caused by mint honey). In some cases, the amount of nuclear and proteic material, evaluated by spectrophotometric readings, if also related to the honey’s biofilm inhibitory activity, let us hypothesize a defective capacity of building the biofilm scaffold or bacterial membrane damage or an incapability of producing them for the biofilm scaffold. The prebiotic potentiality of the honey was assessed on *Lacticaseibacillus casei* Shirota, *Lactobacillus gasseri*, *Lacticaseibacillus paracasei* subsp. *paracasei*, and *Lacticaseibacillus rhamnosus* and indicated their capacity to affect the whole probiotic growth and in vitro adhesive capacity, as well as the antioxidant and cytotoxic abilities, and to inhibit, mainly in the test performed with the *L. casei* Shirota, *L. gasseri*, and *L. paracasei* supernatants, the immature biofilm of the pathogens mentioned above.

## 1. Introduction

For thousands of years, honey has been considered a natural remedy for various health conditions, so it has been used in traditional medicine and is still an important basis for folk medicine. Honey is a natural source of carbohydrates, which can provide a quick energy boost. Its vitamin content, polyphenols, and minerals can also help improve overall health [1]. In the gut, honey effectively reduces the inflammatory process and positively affects its health status, acting on the gut microbiome [2].

Thus, also through the presence of prebiotic compounds, honey can enhance and increase the population of positive bacteria, such as lactobacilli and bifidobacteria [2], which can also counteract the concurrent presence of peroxides present in the honey through an increase in the expression of some antioxidant enzymes, such as superoxide dismutase, heterologous non-heme catalase, glutathione peroxidase, glutathione S-transferase, and glutathione reductase, which are important enzymatic defense systems against oxidative stress [3,4,5]. The concurrent presence of prebiotics peroxides and low water activity creates unsuitable conditions for the growth and development of different pathogenic microorganisms [6].

The mechanisms through which honey acts against pathogens can be multiple. Honey, for instance, can act by modifying some properties of bacteria, such as its hemolytic activity (which is an essential parameter for some bacteria, such as *Escherichia coli*, *Staphylococcus aureus*, and *Pseudomonas aeruginosa*); it can also modify the hydrophobicity of the bacterial cell, thus affecting its capability to adhere to the human cell [7]. Honey can also act in inhibiting or at least reducing the capacity of pathogens to form biofilms, complex communities of microorganisms including bacteria growing on surfaces and embedded in a self-produced matrix of extracellular polymeric substances, or act when this is mature and lead to genotypic and phenotypic modifications of the bacterial cell/community, which determine an increase in the bacterial virulence. [8]. Thus, from a microbiological point of view, honey can carry its beneficial effects on a broad spectrum. The biological properties of honey, including its antimicrobial activity, also depend on its origin [9], the geographical area, and the flower (or flowers in multi-floral honey) from which the final product is made [10]. It can also occur that although two flowers belong to the same species or to the same family, the biological properties of the derived products, such as honey, could be different [11].

Most human pathogenic bacteria causing wound infections, such as *Pseudomonas aeruginosa*, *Staphylococcus aureus*, *Escherichia coli*, and *Streptococcus pyogenes*, are sensitive to honey [12,13]. Indiscriminate use of antibiotics has led to the emergence of multidrug-resistant bacterial strains, a severe threat to public health. Given the increased antibiotic resistance developed over the years by bacteria, the scientific world is in a frantic search for new substances, even of natural origin, which can somehow make up for the weakness of conventional antibiotics, which are not always effective against certain human pathogenic bacteria. Therefore, alternative antimicrobial strategies such as plants and plant-based products, including honey, have currently received more attention [14,15].

Some of the most produced types of honey come from plants belonging to the Lamiaceae family, one of the most widespread plant families in the Mediterranean area. Lamiaceae includes around 250 genera (including *Ocimum*, *Satureja*, *Mentha*, *Salvia*, *Thymus*, and *Origanum*) and almost 7000 species [16,17,18,19]. The plants belonging to this family are widespread, especially in the Mediterranean basin, in the degraded areas of the Mediterranean scrub, and sandy, calcareous, and rocky soils. Plants of the Lamiaceae family have been studied for their biological properties, including the impact they can have on pathogenic microorganisms [20]. The genus *Salvia* has well-documented bacteriostatic and bactericidal properties, as well as antibiofilm properties, against different microorganisms, including *Porphyromonas gingivalis* [21]. *Mentha* × *piperita* (as an essential oil) is active against *Chromobacterium violaceum* but not against *P. aeruginosa*. *Thymus vulgare* (as an essential oil) showed valuable effectiveness in fighting the biofilm of *C. violaceum* and *P. aeruginosa* [22]. Some authors also reported the potential positive effect Lamiaceae can have on useful microorganisms and their influence on the microbiome (prebiotic effect) [23]. The study of Chassagne et al., performed on many Lamiaceae, indicated that there may be often an important overlap between food and medicine in traditional medical practices, given the easy accessibility of food plants [24]. Previous works reported the antibiofilm activity of the honey obtained from some Lamiaceae flowers. Bourkraa et al., for instance, evaluated the synergistic effect of monofloral honey and essential oil from *Origanum* against *P. aeruginosa* [25]. Imtara et al. [26] investigated the antibiofilm activity exhibited by some kinds of honey, including that obtained from *Thymus*. The antibacterial, prebiotic, and postbiotic effects of monofloral honey were substantiated by Fratianni et al. [27], who analyzed the behavior of pathogenic and probiotic strains in the presence of Fabaceae honey. Due to the wide distribution of Lamiaceae plants and the concurrent scarcity of the evaluation of some biological properties of Lamiaceae honey, our work investigated the antimicrobial and prebiotic properties of basil, mint, oregano, rosemary, savory, and thyme honey. The potential antimicrobial action, assessed against the pathogens *Acinetobacter baumannii*, *E. coli*, *L. monocytogenes*, *P. aeruginosa*, and *S. aureus*, took into consideration certain parameters: the capacity of the honey to influence the hydrophobicity and hemolytic activities of the above-mentioned pathogens, as well as the capability of the honey to act on immature and mature biofilm through the measurement of nucleic acids and proteic material. Using *Lacticaseibacillus casei*, *Lactobacillus gasseri*, *Lacticaseibacillus paracasei* subsp. *paracasei*, and *Lacticaseibacillus rhamnosus* as models, we also assessed the in vitro prebiotic property of the honey through the evaluation of its capacity to affect their growth and in vitro adhesive capacity, as well as the antioxidant and cytotoxic abilities. The supernatants of the growth of the probiotic were also tested to evaluate their capacity to inhibit biofilm formation and fight mature biofilms of the pathogens mentioned above.

## 2. Results and Discussion

Our study aimed to evaluate the potential of six types of honey obtained from medicinal plants—basil, mint, oregano, rosemary, savory, and thyme—to act against some pathogens, two of which belong, according to the WHO classification [28], to the critical group (*A. baumanni* and *E. coli*), and two belonging to the high group (*P. aeruginosa* and *S. aureus*). We also considered using *L. monocytogenes*, although it is not part of any of the three groups mentioned above, because its presence indicates severe food poisoning. A former study supported the potential that different kinds of honey can exert on hazardous pathogens [29]. In continuing such an investigation and considering other types of honey obtained from medicinal plants, we did not make the presumption that honey alone could act against these pathogenic strains. More simply, we tried to demonstrate that the presence of honey in the diet might support our body to fight the antimicrobial resistance of such categories of pathogens. Since these types of honey contain a plethora of antibacterial and prebiotic compounds, we could hypothesize that their multi-compositional presence may point to more than one type of cellular modification with an ultimate inhibition of pathogenic bacteria or, in contrast, create the best situation/environment for the growth of probiotics and the expression of their functional properties. Thus, our study was conducted in two directions. On the one hand, we evaluated the effect of six types of honey on certain characteristics of the pathogenic strains, such as hydrophobicity, hemolytic activity, biofilm, and the impact on bacterial DNA and proteins. Concurrently, we considered their prebiotic effect on four probiotic strains. In this case, we evaluated the influence that honey (which substituted glucose in the common MRS growth medium) could have on probiotic growth, antioxidant activity, and hydrophobic capacity, that is, their potential ability to adhere to the intestinal epithelium. The supernatants of the probiotic cultures were used to evaluate the postbiotic effect on model cell lines and the antibiofilm impact on the five pathogens above, indicated in such a way as to try to gain a broader perspective of the potential that honey can exert to limit, in one way or another, the virulence of pathogens. The results are shown in the following tables and figures.

### 2.1. Action of the Honey on Pathogenic Strains

#### 2.1.1. Minimal Inhibitory Concentration

The assessment of the Minimal Inhibitory Concentration (MIC) let us determine the concentration of the honey to be used in all the experiments performed in our study. To evaluate the MIC, we used a range of honey concentrations ranging from 10 to 50 μg/mL. The MIC values are reported in Table 1. Such values were then used to perform all the other experiments.

#### 2.1.2. Hydrophobicity

Cell surface hydrophobicity (CSH) is an essential cellular biophysical parameter that affects microorganisms’ cell–cell and cell–surface interactions and can impact their virulence and biofilm formation. The adhesive strength of a single cell is determined by the number of contact-forming macromolecules and the strength of each binding site. The composition of surface macromolecules and important adhesion parameters, such as the bacterial contact area to solid surfaces, are highly individual cell properties [30,31,32]. A CSH 152 study demonstrated that the adhesion of some beneficial and pathogenic bacteria, such as *Lactobacillus plantarum*, *Streptococcus mutans*, and *S. aureus,* to hydrophobic surfaces is about one order of magnitude stronger than to hydrophilic surfaces [33]. Some studies reported that honey also exerts antibacterial action by modifying the hydrophobicity characteristics of its membrane [34,35]. In our experiment, we evaluated whether the presence of honey during the growth of the five pathogenic strains could somehow influence the hydrophobicity characteristics of their cells and, therefore, their potential ability to adhere to the intestinal epithelium. The results are shown in Table 2.

#### 2.1.3. Hemolytic Activity of Pathogens Grown in the Presence of Honey

Hemolytic activity is a recognized virulence factor impacting bacterial pathogenesis [36]. Hemolytic bacteria, through the action of the hemolysins, may also provoke many types of infection. The hemolytic activity of hemolysins is due to the formation of pores on the erythrocyte surface, disrupting the membrane integrity [37]. Several bacteria increase their virulence through hemolytic activity too, including *A. baumannii* [38], *L. monocytogenes* [39], *P. aeruginosa* [40], and *E. coli* [41]. To analyze at least some aspects of the antimicrobial potential exhibited by these kinds of honey, we also tried to evaluate whether the presence of honey in the culture broth of the five pathogens modified their hemolytic activity in a test conducted using defibrinated sheep blood. The results are shown in Table 3.

The strains exhibited different behaviors depending on the type of honey added. The hemolytic activity of *A. baumannii* was 42.5% in conventional conditions. Basil honey and savory honey dropped its activity to 29.3% and 32.5%, respectively. Oregano honey (hemolytic activity = 37.6%), mint (8.3%), and mainly rosemary honey (40.1%) had a slight inhibitory effect on the hemolytic capacity of the bacterial strain. In contrast, thyme honey enhanced the hemolytic action of *A. baumannii*, reaching 44.6%, a 2% increase compared to the control. The hemolytic activity of *E. coli*, already lower (11.6%) compared to *A. baumannii*, decreased markedly in the presence of rosemary honey (8.1%) and oregano honey (7.9%), and more significantly in the presence of basil honey—which reduced it by approximately 50% (7% vs. 11.6%)—and above all, mint honey, which contributed to significantly lowering the hemolytic efficacy by 75%, decreasing the value from 11.6% to 2.8%. In the test performed on *L. monocytogenes*, which had shallow hemolytic activity *per se* (3.7%), the activity was further lowered when the pathogen was in contact with rosemary and savory honey, but above all, when in contact with thyme (1.5%) and oregano (1.2%) honey. Instead, mint honey, and especially basil honey, increased its hemolytic effectiveness up to five times. The presence of honey contributed to lowering the hemolytic activity of *P. aeruginosa*, bringing it from a standard value of 23.6% up to 10.4% when the strain was incubated in the presence of oregano honey and 9.0% when the incubation was performed with savory honey. *S. aureus* demonstrated unusually low baseline hemolytic activity (7.2%). The presence of honey determined lower hemolytic efficacy when the strain was incubated with basil (3.1%), oregano (4.5%), savory (5.1%), and thyme (5.1%) honey. In contrast, rosemary increased its hemolytic action, bringing its value from 7.2% to more than double (17.7%). Our results corroborated previous studies reporting the potential hemolytic inhibitory action exhibited by the honey on different pathogenic bacteria. Ramon-Serra et al. [42] observed that, in some cases, the hemolytic activity, particularly of *E. coli* and *S. aureus*, decreased in contact with the honey’s polyphenols. Brown et al. [43] reported that the use of sublethal concentrations of manuka honey on different isolates of *Staphylococcus pseudintermedius* significantly reduced hemolysin activity in half of the *S. pseudintermedius* isolates, indicating a strain-specific mode of action. In *S. aureus*, β-hemolysin production promotes efficient skin colonization [44]. It is also believed to trigger host cell cytotoxicity, act as a biofilm ligase, and give a selective advantage to those strains producing it [45]. They thus suggested the capability of honey to diminish β hemolytic activity and subsequently lower the capability of *Staphylococcus* to colonize host cells and cause cytotoxicity. Therefore, the effect on the hemolytic activity of pathogens can be a clear indication of the capacity of the honey to fight the virulence of pathogens, even if, in some cases, it is not acting on bacterial growth [46]. The study performed by Salosso et al., conducted on *Vibrio alginolyticus* using the left forest honey, corroborated the hypothesis that honey can act against several pathogens, also decreasing their hemolytic activity [47].

#### 2.1.4. Antibiofilm Activity of the Honey

For thousands of years, honey has received considerable attention for its therapeutic properties, mainly due to its capacity to act against microorganisms [48]. In recent years, such attention has increased and was also addressed to investigate the potentiality of honey in inhibiting or limiting the virulence of pathogens by blocking the plethora of events giving rise to the biofilm, which is, as well-known, the condition that leads to the bacterial cells not only providing themselves with a protective shield against the defense mechanisms of the human body or antibiotics but also changing their metabolism and increasing their virulence. Monofloral bioactive honey is extensively pursued and consequently estimated, as seen in the rising worldwide demand to identify the best candidates for specialist pharmaceutical honey, in addition to manuka (*Leptospermum scoparium* J. R. Forst & G. Forst) honey, recognized as one of the most famous worldwide [49].

Through the CV test (Table S1), we evaluated the potential of the six types of honey obtained from medicinal plants to inhibit the adhesion process of the five pathogenic bacteria and act on their mature biofilm. Through the MTT test (Table S2), we then monitored whether the presence of honey in the culture broth could inhibit sessile cell metabolism. Finally, through a spectrophotometric reading, performed at 260 and 280 nm (Table 4), we intended to see if the presence of honey at the highest concentration tested, added at time zero and after 24 h in the CV and MTT tests, could have caused any damage to the bacterial cells, with consequent leakage of nuclear material (reading at 260 nm) or proteins (reading at 280 nm).

##### Action of the Honey on Bacterial Biofilm

The addition of honey at the beginning of the bacterial incubation inhibited the bacterial adhesion process and, therefore, biofilm formation, more evidently only against *A. baumannii* and, above all, against *S. aureus*. This last was sensitive to the presence of all six types of honey, determining the inhibition of its immature biofilm.

Mint, oregano, and rosemary honey were the most active against the immature biofilm of *S. aureus*, causing inhibition percentages higher than 40% (45.65%, 41.31%, and 56.48%, respectively), but the inhibition was evident even in the presence of the smaller quantity (4 μg/mL) of mint (35.31%) and rosemary honey (48.07%). The effect of savory honey was similar, independent of the concentration of honey used, with percentages of inhibition of 38.01% and 38.21%. Thyme honey inhibited the immature biofilm (31.52%) when tested at the highest concentration, albeit less effectively; basil honey was also capable of acting on the immature biofilm of *S. aureus* (22.55%). *A. baumannii* was also sensitive to the presence of all kinds of honey, albeit less markedly. Rosemary (inhibition = 32.15%) and thyme (inhibition = 20.40%) were the most active types of honey in inhibiting its immature biofilm. Rosemary was the only one to act by inhibiting the immature biofilm of *P. aeruginosa* (16.96%) and, together with savory, the only one to limit the *E. coli* immature biofilm (9.85% and 23.93%, respectively). *L. monocytogenes* was insensitive to the presence of all six types of honey, except for the weak inhibitory action (3.09%) exerted by thyme honey.

The situation was completely different when we added the honey to the bacterial culture after 24 h, thus on mature biofilm (indicated in Table S1 as “24”). The bacterial strains’ resulting resistance to the presence of honey added at the beginning of their growth was, conversely, much more sensitive, and in many cases, we observed much higher inhibition percentages. For example, honey inhibited the mature biofilm of *L. monocytogenes*, causing % inhibition no lower than 35.69% (with 8 μg/mL of basil honey). Interestingly, the honey incapable of blocking the immature biofilm of *L. monocytogenes* instead managed to act on its mature biofilm, inhibiting it by 42.22% (oregano), 61.72% (rosemary), 68.85% (thyme), and even 81.62% (mint). The test evidenced the almost ineffective inhibitory action exerted by basil and mint honey on the mature biofilm of *E. coli*. In contrast, the other kinds of honey inhibited its mature biofilm, with percentages ranging—in the test conducted with 8 μg/mL—between 31% (oregano honey) and 63.95% (savory honey). A greater propensity to act against the mature biofilm was also observed when we added the honey to the *P. aeruginosa* culture broth. Rosemary honey confirmed, or rather accentuated, its inhibitory action, going from a value of 16.96% to 60.42%; oregano honey, ineffective on *P. aeruginosa* immature biofilm, was instead capable of exerting an inhibitory action on its mature biofilm (54.41%). Although less effective than those mentioned above, mint and basil honey could still inhibit the mature biofilm of *P. aeruginosa*, with inhibition percentages equal to 15.12% and 9.45%, respectively. Savory and thyme honey, however, confirmed their ineffectiveness against this strain. *S. aureus* also proved sensitive to the presence of honey in the test conducted on mature biofilm. Mint honey was the most effective (80.32%); in any case, the inhibitory action exhibited by the other types of honey was never lower than 51.07% (rosemary honey), even reaching inhibition percentages equal to 67.38% (basil honey) in the tests conducted with 8 μg/mL. Indeed, in the 4 μg/mL test, mint honey exerted an inhibitory action equal to 55.25%. *A. baumannii*, although resistant to honey tested at the lowest concentration, was sensitive when the tests were performed at the highest concentration, except for oregano and savory honey, which were completely ineffective. The other kinds of honey exhibited similar inhibitory action, ranging between 50.11% (thyme) and 60.27% (basil).

##### Action of the Honey on Sessile Bacterial Metabolism

The analysis of the inhibition percentages, calculated in the MTT test performed on immature and mature biofilm (Table S2), highlighted that the different kinds of honey were ineffective at inhibiting the bacterial cells present within the immature biofilm of *A. baumannii*, *E. coli*, and *L. monocytogenes*.

The honey could instead act on the bacterial cells’ metabolism within the immature biofilm of *P. aeruginosa* and *S. aureus*. In the case of *P. aeruginosa*, all kinds of honey seemed to act against its cells. Thyme (inhibition = 50.26%), rosemary (inhibition = 44.25%), savory (inhibition = 35.27%), and basil honey (inhibition = 34.05%) were more effective in acting on the metabolism of *P. aeruginosa* bacterial cells present within the immature biofilm. Thyme honey was very effective even at the lowest concentration, where we recorded an inhibition percentage of 48.42%. *S. aureus*, which had also shown itself to be the weakest against the action of honey in the crystal violet test conducted on immature biofilm, exhibited an albeit greater resistance to the action of honey on its metabolism; however, the different kinds of honey were all able to act on the metabolism of its sessile cells, with inhibition percentages ranging between 8.77% (mint) and 53.33% (oregano). The situation was completely different when we analyzed the effect of honey on the metabolism of the sessile cells of the mature biofilm. In this case, honey proved to be completely ineffective. We observed certain inhibitory action by thyme honey when added at a concentration of 8 μg/mL against the sessile cells of *L. monocytogenes*, which were also weakly sensitive (inhibition = 8.81%) to the presence of rosemary honey. The action of mint honey against the metabolism of the sessile cells of the mature biofilm of *S. aureus* was practically imperceptible (inhibition = 1.45%). The data are particularly encouraging. In fact, they show that honey is capable of acting not so much on the immature biofilm, or at least not only, but they can block, as we have seen in some cases with very high percentages of inhibition, the mature biofilm, a condition that determines, for all the modifications of the bacterial cell within the biofilm, an increase in its virulent power, and therefore a more complex difficulty, on the part of conventional drugs, to its eradication. Based on the results of the MTT test, we could say that the honey could act in different ways and that the action on metabolism is not always the principal effect.

##### Evaluation of the Amount of Extracellular Bacterial DNA and Proteins

The bacterial membrane offers outstanding protection. Thus, the presence of nucleic acids and proteins in the culture medium is a clear sign of bacterial membrane damage; however, it can be also an indicator of the capacity of the bacteria to build the polymeric biofilm scaffold. Thus, the measurement of the absorbance of the released nucleic acids and proteins at 260 nm and 280 nm, respectively, was taken into consideration to evaluate if the presence of honey could affect the building of the biofilm or potential damage to the bacterial membrane’s integrity. The culture medium was obtained and filtered in two distinct moments, the first after 48 h of incubation of the bacteria grown in the presence of honey added at time zero and the second after 48 h of cell growth, when honey was added after 24 h after the initial incubation (when bacteria presumably formed a mature biofilm). The measure of absorbance was carried out on the culture medium obtained after centrifugation and filtration, ensuring the accuracy of our results. The results are shown in Table 4 (release of nucleic acids) and Table 5 (release of proteic material).

Generally, all kinds of honey, when added at time zero, determined a substantial decrease in the amount of extracellular nucleic acids. The addition of basil, mint, and savory honey induced a marked decrease in the amount of extracellular nucleic acids, and in several cases, such values were completely zeroed (Table 4). The situation was different when the honey was added 24 h after the beginning of growth. In fact, while some kinds of honey such as basil, mint, and thyme continued to act, noticeably decreasing the amount of nucleic acid present in the culture medium of the pathogens, the presence of other honey such as oregano, rosemary, and savory caused a marked modification of the absorbance unit values, which increased from 2.38 absorbance units (*E. coli*, control) to 3.88, 3.89, and 4.98 (*E. coli* grown in the presence of oregano honey, rosemary, and savory honey, respectively) and 5.02, 4.99, and 5.08 (*L. monocytogenes* grown in the presence of oregano, rosemary, and savory honey, respectively). The decrease in the absorbance units’ values was evident when the test was conducted on *P. aeruginosa*, independent of the type of honey considered. The spectrophotometric analysis—performed on the growth supernatant after 48 h of bacterial growth, but with the addition of the honey 24 h after the initial incubation—highlighted, for many aspects, the opposite behavior to what we observed in previous measurements. We can observe that the absorbance values are almost always zeroed compared to the control. From a comparison of the data in Table 4 and Table 5, we could hypothesize that when we added the honey at time zero, in some cases, this determined an increase in the number of extracellular proteins. If we included the honey after 24 h, we observed, in some cases, an increase in the extracellular nucleic acid amount.

The presence of extracellular material could represent, as indicated above, the capacity of bacteria to produce the scaffold for the building of the biofilm or, in contrast, damage to the cells induced by the presence of honey in the culture medium. To explain one of these hypotheses or the other, we should compare the results shown in Table 4 and Table 5 with the biofilm inhibitory activity data shown in Tables S1 and S2. In general, basil honey does not seem to have had much influence, if added at time zero, on the biofilm inhibitory capacity of pathogenic strains, as demonstrated both by the percentage of inhibition (zero) and the absorbance values at 280 nm, which were, in the case of *P. aeruginosa*, much higher than the relative control. In the case of *A. baumannii* (% inhibition = 12.63%, Table S1), the fact that an absorbance value at OD 280 nm = 2.8 was observed, which was higher than the control, could lead to bias not only towards the production of volunteer proteins for the biofilm scaffold but also damage to the bacterial cell produced by the presence of basil honey on the cell membrane compared to the control (absorbance units 1.60 vs. 1.02, respectively). The very high absorbance value (7.50) recorded by the culture medium of *P. aeruginosa* grown in the presence of basil honey could suggest weakness of the bacterial cell showing an altered metabolism (34.05%, Table S2), which perhaps caused damage to the cell membrane. In contrast, the zero absorbance values at 260 and 280 nm observed for *S. aureus*, associated with the biofilm inhibition value (22.55%, Table S1), would suggest that basil honey had somehow blocked the production of nuclear and protein material for the biofilm scaffold.

Mint honey exhibited poor biofilm inhibitory activity against *E. coli* and *L. monocytogenes*, as evidenced by the zero-inhibition value (Table S1) and the high absorbance values found in the spectrophotometric reading at 280 nm, which would indicate the presence of proteins, especially for the polymeric scaffold of the biofilm. In the case of *P. aeruginosa,* we recorded an inhibition value = 0 and a decrease in absorbance units at 260 and 280 nm, which started, however, in the control with already low values. *A. baumannii* instead shows a slight decrease in its biofilm-producing capacity (inhibition = 11.71%, Table S1), and a decrease of approximately 50% in the absorbance values at 260 and 280 nm, which would suggest inhibitory action on the ability of the bacterium to produce extracellular material. The fact that *S. aureus* suffered significant inhibition of its ability to form the biofilm (inhibition = 45.65%, Table S1) and that it did not show the presence of nuclear or extracellular protein material in the culture medium suggests that, in this case, the presence of mint honey had somehow blocked the production of extracellular material for the biofilm scaffold. Rosemary honey determined the inhibition of the biofilm activity on almost all bacterial strains except *L. monocytogenes* (Table S1). The highest inhibition values were found when we included rosemary honey in the culture medium of *A. baumannii* (32.15%, Table S1) and *S. aureus* (56.48%, Table S1). Now, in these two cases, the absorbance values observed at 260 nm and 280 nm suggested a clear decrease in the production of extracellular material of nuclear origin and, in the case of *A. baumannii*, damage to the cell membrane, as demonstrated by the large amount of protein material recorded in the culture medium (absorbance units = 7.38) compared to the control. Savory honey, which induced an inhibition in biofilm formation vs. *A. baumannii*, *E. coli*, and *S. aureus* (Table S1), seems to have decreased the production of extracellular material (Table 4 and Table 5) rather than induced damage to the cell membrane, while, in the case of *P. aeruginosa*, the efficacy of savory honey resulted, as we have seen, mainly in inhibitory action on the metabolism of its cells included in the biofilm (44.25%, Table S2), which perhaps also determined damage as demonstrated by the higher absorbance value compared to the control, as indicated in Table 4 and Table 5. Thyme honey, which acted essentially on the biofilm-forming capacity of *A. baumannii* and *S. aureus* (Table S1) on the metabolism of the cells present in the biofilm of *P. aeruginosa* (Table S2), in the first two cases, likely limited the capacity to form extracellular material. In contrast, in the case of *P. aeruginosa*, it would have caused damage to the cell membrane (Table 4 and Table 5).

The findings of this study have practical implications. The addition of honey after the first 24 h significantly decreased the biofilm effectiveness of the bacteria (Table S1), without causing evident damage to their metabolism (Table S2). This suggests potential applications in biofilm control. The absorbance data shown in Table 4 and Table 5, where a consistent decrease in the values at 280 nm compared to the control was observed, further support these implications.

With a few other exceptions, honey could inhibit the mature biofilm of the five pathogenic strains, even with very high percentages (Table S1). Such action could be linked to an incisive action on the ability to produce extracellular material. In some cases, for example, when honey was added to the culture medium of *E. coli*, it was not always able to inhibit the biofilm (basil honey and mint honey). Here, the comparison with the absorbance data of Table 4 and Table 5 suggests the effect of honey on the ability to produce extracellular material, which did not completely prevent the bacteria from stabilizing their mature biofilm. In other cases, honey may have acted either by causing damage to the cell membrane (*L. monocytogenes*) or by preventing a sufficient production of extracellular material, especially nuclear (oregano honey vs. *P. aeruginosa*, Table 4). *S. aureus*, which showed a strong sensitivity to the presence of all types of honey (Table S1), always presented null or almost null absorbance values at 260 and 280 nm. This would indicate that the action of honey was significant in the blocking of the production of extracellular material.

Our study corroborated previous studies, which, from a purely pre-clinical point of view, demonstrated that honey can inhibit immature biofilm formation and reduce the established mature biofilms. Honey can, therefore, exert antibacterial activity against several multidrug-resistant bacteria [29,50]. Our study goes even further, as it demonstrates that these six types of honey are not only able to inhibit the biofilm upstream in many cases but also often act on the mature biofilm and, in some cases, damage bacterial cells, with the release of cellular material (nucleic acids and proteins), as indicated by the data from both the CV test and the spectrophotometric readings conducted at 260 nm and 280 nm. At the level of cellular physiology, the presence of honey determines a modification of some properties, generally indicative of increased virulence, such as hydrophobicity and hemolytic activity. Given the great worldwide distribution of the Lamiaceae and the significant applications of their honey, various studies have demonstrated that their honey could be considered very interesting from the microbiological point of view, also through the capability to affect the growth and virulence of pathogenic bacteria [51,52].

**Figure 1:**
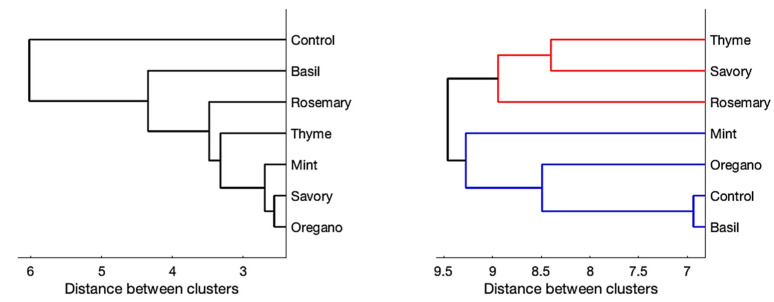
(**left**). Dendrogram of the Lamiaceae kinds of honey obtained using a UPGMA clustering algorithm, taking into account the results shown in Table 2 and Table 3. (**right**). Dendrogram of the Lamiaceae kinds of honey obtained using a UPGMA clustering algorithm, taking into account the results shown in Tables S1 and S2.

After standardization, the data in Table 2 and Table 3 were clustered using an unweighted average distance binary (UPGMA) clustering algorithm; the dendrogram is shown in Figure 1 (left). All types of honey showed significantly diverse behavior with respect to the control. Oregano, thyme, mint, savory, and rosemary honey were similar, with basil deviating slightly. Applying the same approach with respect to the data reported in Tables S1 and S2 (Figure 1, right), we can quickly identify two clusters: the first red-colored cluster containing thyme, savory, and rosemary honey and the second cluster (blue-colored) formed by oregano, mint, basil, and the control. In particular, basil showed results very close to the control.

### 2.2. Prebiotic Effect of the Honey

Honey is capable of exerting a positive impact on beneficial bacteria [29,53]. It may affect their growth and the hydrophobicity properties of their cells and increase their antioxidative properties [27,54]. It is noteworthy that honey has an impact on lactobacilli’s probiotic activity, including its antimicrobial activity against pathogenic and unwanted bacteria [55]. We investigated the effect that the six kinds of Lamiaceae honey could exert on some biological characteristics of probiotic bacteria. Specifically, we evaluated the impact of honey used instead of glucose in the MRS culture broth on lactobacilli growth, in vitro adhesion, and antioxidant activity. The effect of the LAB growth supernatants was also evaluated on two cell lines and against the five pathogenic strains to assess the cytotoxicity and antibiofilm activity, respectively.

#### 2.2.1. Effect of Honey on the Probiotics’ Growth

To evaluate the potential growth-stimulating effect of the six types of honey, we replaced glucose with honey in the MRS formula. The results are shown in Table 6. The data relating to the spectrophotometric measurements conducted at a wavelength of λ = 600 nm highlight that the type of honey influenced the growth of probiotics in a different way. *L. casei* and *L. rhamnosus* growth was always positively affected by the presence of honey. In the case of *L. casei*, the influence was honey-dependent. Thus, apart from mint honey, which determined a weak increase in bacterial growth, the other kinds of honey influenced the growth of this probiotic more markedly, with ∆, calculated with respect to the control, ranging from 8% (basil), 15.4% (thyme), 18.56% (savory), 18.95% (oregano) up to 26.1%, when L. casei was grown in the presence of rosemary honey. Although always positive, the influence of the various types of honey on the growth of *L. rhamnosus* was less variable and less marked. In fact, apart from oregano, whose presence determined an increase equal only to ∆ = 3.60% compared to the control, honey caused a somewhat similar increase in bacterial growth, ∆ ranging between 8.94% (rosemary) and 11.48% (thyme). Only two types of honey (rosemary and savory) stimulated the growth of *L. gasseri* (∆4.74% and 5.07%, respectively). Conversely, the presence of basil, mint, and thyme honey was weakly inhibitory. Instead, no honey had a growth-stimulating effect on *L. paracasei*.

Our data, although not completely regarding all four strains tested, agree with previous research, which demonstrated a growth-stimulating effect on some probiotic microorganisms. Carvalho de Melo et al. [55] underlined the functional properties of different monofloral kinds of honey on *L. acidophilus* and *Bifidobacterium*. Das et al. [56] ascertained a growth-promoting effect of some *Sesamum indicum* kinds of honey on *L. acidophilus*; similarly, Shamala et al. observed a positive effect of honey produced in a coffee area on the growth of *L. acidophilus* and *L. plantarum* indeed [57]. Our data are in disagreement with what was observed by Kgozeimeh et al. [58], who found a negative effect of honey on the growth of both *L. casei* and *L. rhamnosus*. Our data are also discordant with what was observed by Fratianni et al. [59], who, studying the effect of legume honey on the growth of some strains of lactobacilli, found its stimulating solid action on *L. gasseri*. In contrast, this action turned out to be practically nil when he went to evaluate the effect on the growth of *L. rhamnosus.*

#### 2.2.2. Hydrophobicity of Probiotics

The measurement of cell surface hydrophobicity can indicate probiotics’ capacity to adhere to the intestinal epithelial cells [60]. Adhesion to hydrocarbons like xylene can be reputed as a biochemical marker for adherence to the epithelial cells in the gut. The influence of honey on the growth and in vitro adhesive capacity of the five strains of probiotic bacteria has been analyzed by verifying the difference in their growth under conventional conditions (in MRS broth). Hence, we assessed the cell surface hydrophobicity of *L. gasseri*, *L. casei* Shirota, *L. paracasei*, and *L. rhamnosus* with the organic solvent xylene after 3 h. The results are shown in Table 7.

Unlike microbial growth, honey rarely influences the hydrophobicity of the microorganisms. Each type of honey affected the bacteria differently. Savory honey caused an increase in the *L. gasseri* hydrophobicity, but above all, oregano honey caused an increase in hydrophobicity of that strain equal to 27.26% compared to the control. Basil honey increased the hydrophobicity of *L. rhamnosus* (1.30%) but especially of *L. paracasei* (13.42%). Considering all the results, we could assume that *L. paracasei* (13.42%) and *L. gasseri* (27.26%) were the bacteria most influenced by the presence of honey. Rosemary honey increased the hydrophobicity (9.28%) of *L. casei*, which was also influenced, albeit in a barely perceptible way (2.4%), by the presence of thyme honey. Honey, therefore, in many cases confirmed its good prebiotic action [61], as it can act positively not only on the growth but also on the hydrophobicity of lactic acid bacteria. We could say that, in some cases, a certain type of honey caused an increase in bacterial growth and cellular hydrophobicity. For instance, rosemary honey and thyme honey not only caused an increase in *L. casei* growth but also its hydrophobicity. The increase in hydrophobicity found for *L. gasseri* (27.26% in the presence of oregano) and *L. paracasei* (13.2% in the presence of basil) did not correspond to a concomitant increase in bacterial growth. In contrast, an increase in bacterial growth (which occurred when we cultured *L. casei* in the presence of oregano or when we incubated *L. rhamnosus* in the presence of mint) did not correspond to a parallel improvement in performance of cells hydrophobicity. In other cases, we found a null effect exerted by honey both in terms of growth and hydrophobicity. Our data partially agree with what was reported by Fratianni et al. [27], which often observed an inverse correlation between the influence of honey on bacterial growth and its hydrophobicity in some cases.

#### 2.2.3. Antioxidative Properties of Probiotics

Reactive oxygen substances (ROS) can damage the cells, contributing to cardiovascular, chronic inflammatory, cancer, and neurodegenerative diseases [62]. Although in normal conditions our body can counteract such situations by synthesizing antioxidant enzymes and specific compounds that, jointly with the food antioxidants, assemble a biological antioxidant barrier, in some conditions the defense system could be lacking. Thus, we need to increase the antioxidant defenses to preserve our health and fight or prevent diseases related to antioxidant deficiency. In this direction, an interesting approach is the possibility of exploiting probiotics’ antioxidant activity, which can counteract the oxidative stress in the host, thus determining a decrease in the risk of accumulation of ROS [63]. Lactic acid bacteria (LAB) can generally resist ROS, and the inclusion of probiotics in our diet can also defend normal liver functions [64]. Some studies, such as that of Amaretti et al. [65], ascertained such properties for some lactobacilli, and Won et al. demonstrated the antioxidant activity of *L. paracasei* [66]. Bee honey has always fascinated the scientific community as it is a natural dietary antioxidant [60,67]. Recently, Fratianni et al. studied the effect that some types of honey originating from leguminous plants could exhibit when added to the growth medium in place of glucose on some probiotic strains’ antioxidant capacity through different tests [27]. Starting from such considerations, we have evaluated the influence of the six types of herb honey on the antioxidant capacity of the probiotics *L. paracasei*, *L. gasseri*, *L. casei* Shirota, and *L. rhamnosus*. The results are shown in Table 8.

The ILAP test is mainly used to measure lipid oxidation and antioxidant activity in biological systems. In the test, we used linoleic acid as a substrate because its degeneration at the cellular level causes damage at the level of primary metabolites (structural proteins and enzymes). LAB exhibited a different behavior in the ILAP test; once again, basil honey was the most effective, acting beneficially on almost all probiotic strains, with values always higher than control, and in some cases rising noticeably. When added to the growth medium of *L. rhamnosus*, basil honey increased the inhibitory effect on lipid peroxidation, with values nine times higher than the control (11.7% vs. 1.30%, respectively, in the test with *L. rhamnosus*) and even 15 times more when added to the growth medium of *L. paracasei* (18.3% vs. 1.30% of the control). It is also important to underline the positive effect on *L. gasseri*, for which ILAP percentages increased from 1.30% up to 18.3%. The inclusion of mint honey in the growth medium caused a similar behavior: this honey caused a decrease in the ILAP activity when added to the growth medium of *L. casei* Shirota (50% less than the control). In contrast, ILAP percentages increased when the honey was added to the growth medium of *L. rhamnosus*, *L. paracasei*, and *L. gasseri* (ILAP = 8.8%, 15.9%, and 17.0%, respectively). Oregano, rosemary, and savory exhibited a weaker positive activity. Oregano honey ameliorated the percentage only in the case of *L. paracasei* (15.7% vs. 11.9% of the relative control) and *L. rhamnosus* (6.6% vs. 1.30% of the relative control). Rosemary and savory honey acted by ameliorating the inhibitory capacity only of *L. rhamnosus* (12.4% and 3.9%, respectively). *L. casei* was insensitive to the presence of the honey, so its inhibitory capacity on lipid peroxidation decreased markedly in the case of thyme honey and oregano honey from 15.8% to 5.3% and 5%, respectively. The OH scavenging activity (expressed as a percentage) confirmed, once again, the positive effect exhibited by the presence of basil honey, which caused an increase in the activity from 37.9% (*L. paracasei*) up to 57.5% (*L. rhamnosus*) and 61.3% (*L. gasseri*). We should underline that, in general, almost all honey could increase the radical scavenging activity of the probiotic strains. Basil, mint, and thyme honey increased the OH radical scavenging of all four LABs. Rosemary honey was ineffective only when added to the growth medium of *L. casei* Shirota. Savory honey acted positively on the OH radical scavenging activity of *L. paracasei* and *L. rhamnosus*. Oregano honey was the weakest honey, acting positively only when added to the growth medium of *L. rhamnosus*. In the test to evaluate the CFS reduction activity (which expresses the results in terms of cysteine equivalents), we observed a positive effect of oregano honey on *L. casei* Shirota (with an increase in mM of cysteine Eq from 1.58 to 1.77), and *L. rhamnosus* (with an increase in mM of cysteine Eq from 1.02 to 2.27). Basil honey was particularly active in the test carried out on *L. paracasei* (from 1.38 to 2.11 mM cysteine Eq) and *L. rhamnosus* (from 1.02 to 2.01 mM cysteine Eq). Rosemary and mint honey were active only when added to the growth medium of *L. rhamnosus*; however, they were capable of noticeably increasing the amount of mM of cysteine Eq with respect to the control, with values increasing from 1.02 mM to 2.67 mM (mint honey) and 3.20 mM (rosemary honey).

By comparing the antioxidant activity of probiotics grown in the presence of honey (s) with the corresponding controls (c), we attempted to identify which type of honey may have influenced their antioxidant performances and how. First, unlike what Fratianni et al. [27] saw, not all Lamiaceae honey improved bacterial hydroxyl scavenging efficacy, and honey did not have such a vigorous action. Among the various kinds of honey, thyme honey exerted a more significant positive action than other types of honey in the case of *L. rhamnosus* (s/c = 2.51), *L. paracasei* (s/c = 2.29), and, to a lesser extent, *L. gasseri* (s/c = 1.78). The influence of honey was even less in the CFS test, except for rosemary honey (s/c = 3.13) and savory honey (s/c = 2.74). The influence exerted by Lamiaceae honey in the ILAP test was much more effective, with reference to *L. rhamnosus*. In this case, we started with s/c ratios = 3 and 3.08 when *L. rhamnosus* was incubated in the presence of savory and rosemary honey, respectively, but mainly when it was incubated in the presence of mint (s/c = 6.30), basil (s/c = 9), and rosemary honey (s/c = 9.53). These data differ from those of Fratianni et al. [27], who saw an intense protective action exhibited by legume kinds of honey on *L. gasseri* but not on *L. rhamnosus*. In our study, *L. rhamnosus* seemed to be the most susceptible probiotic strain to the protective action of honey, so much so that, with a few exceptions, the s/c ratio exhibited in the tests was more significant than the s/c ratio calculable from the results shown by the other strains. Our data suggest that, in different ways, the types of honey we consider can inhibit or at least limit the production of oxidant compounds in the intestine. Therefore, through its antioxidant actions, honey may have protective actions in pathologies such as colon cancer. The fact that, in different cases, the LABs increased their capacity to fight the linoleic acid peroxidation is undeniably a motivating sign of the increased functional capacity of probiotics, albeit in vitro.

#### 2.2.4. Cytotoxic Activity of Probiotics

Different studies, with different cell lines and types of honey, or in vivo, on mice and rats, established the potential impact of honey on the prevention, treatment, and progression of cancer [68,69,70]. The anticancer properties of honey can be related to different mechanisms, including apoptosis, stopping the cell cycle, regulating oxidative stress, bettering inflammation, stimulating mitochondrial outer membrane permeabilization (MOMP), and inhibiting angiogenesis [71]. Lactic acid bacteria are a precious source of bioagents that can be used to treat and prevent different types of cancer, including those affecting the breast [72], cervical [73], and colon [74]. Probiotics exhibit a tumor-suppressive effect by producing some apoptosis-inducing compounds [75]. Recently the effect of dietary honey has also been studied on colonic probiotic bacteria in rats [76]. Razan et al. investigated the potential of manuka honey (MH) as an immunomodulatory agent in colorectal cancer (CRC), demonstrating through bacterial 16S rRNA sequencing that oral MH treatment induced unique changes in gut microbiota that may well underlie the IFN-dependent enhancement in tumor immunogenicity [77]. Moreover, various types of honey exhibit anticancer properties against different cancer cell lines. In addition, lactobacilli have been shown to exert cytotoxic effects on several cancer types, although interactions between these beneficial microorganisms and honey have been sparsely investigated, particularly regarding their combined effects on cancer. In a study published in 2020 [78], when used as a carbon source for probiotics, chestnut honey enhanced the in vitro cytotoxic effects of probiotic bacteria against MCF-7 cells. Similarly, it was demonstrated that adding lime honey boosted lactobacilli’s growth and increased cytotoxicity against breast and colon cancer cells [79]. Thus, honey can directly impact human health and provide indirect advantages through beneficial microorganisms. The synbiotic interaction between honey and probiotic bacteria holds the potential for enhancing mutual benefits. Given that synbiotics—combinations of probiotics with other dietary constituents—may confer greater health benefits, investigating the effects of honey on probiotic bacteria such as *L. casei* Shirota, *L. paracasei*, *L. rhamnosus*, and *L. gasseri* is of interest. The evaluation of the in vitro cytotoxic effects of the combination of probiotics and Lamiaceae honey on breast cancer cell line MCF-7 and colon cancer cell line Caco-2 could provide further insights into their potential synergistic effects against cancer. In our study, cell-free supernatants containing secreted metabolites of the probiotics were utilized to assess the antiproliferative activities of the four probiotic strains. We aimed to determine if this combination of probiotics and honey yielded more positive effects than those cultivated on glucose or honey alone. Following the treatment of MCF-7 and Caco-2 cells with different dilutions (1/2, 1/4, and 1/6) of the probiotic cell-free supernatants for 24 h, the antiproliferative activities were measured and are illustrated in Figures S1 and S2. While honey alone did not significantly affect the cell viability of MCF-7 and Caco-2 cells at dilutions of 1/6 and 1/4, the 1/2-diluted supernatants of probiotics grown on honey (specifically mint honey) reduced cell viability by up to 35% against MCF-7 cells. As depicted in Figure S1a,b, *L. casei* Shirota and *L. gasseri* grown in the presence of mint, oregano, and rosemary honey exhibited stronger antiproliferative effects on both cancer cell lines compared to probiotics or honey alone. *L. gasseri* grown with savory honey also demonstrated cytotoxic effects (Figure S1b), while *L. paracasei* (Figure S2a) exhibited these effects with mint, oregano, and savory honey but not with rosemary honey. *L. rhamnosus* (Figure S2b) showed these effects only with mint and savory honey against the MCF-7 cell line. This observation aligns with the research of Celebioglu et al. [61], where *L. acidophilus* grown on lime honey exerted greater anti-proliferative effects on the MCF-7 cell line compared to the Caco-2 cell line. In contrast, thyme honey did not alter the cytotoxic activity of *L. rhamnosus* on either breast or colon cancer cells, as this probiotic alone demonstrated potent cytotoxic effects. Our study revealed that when probiotics are generally cultured in the presence of Lamiaceae honey as a carbon source, the viability of breast and colon cancer cells was significantly reduced compared to probiotics grown on glucose or honey alone. This suggests that incorporating Lamiaceae honey into the bacterial growth medium enhances cytotoxic effects. Honey displays anticancer properties against various cancer cells, but no prior research has explored the potential interactions between beneficial microorganisms and Lamiaceae honey and their combined effects on cancer cells. The cytotoxicity results highlight advantageous interactions between probiotics and honey. Thus, new formulations that include both Lamiaceae honey and probiotics may offer greater benefits compared to using either bacteria or honey alone.

#### 2.2.5. Antibiofilm Activity of Probiotics

The crystal violet test, performed on *L. casei*, first highlighted that the supernatant of the control, i.e., of the bacterium grown in MRS, exhibited a diverse antibiofilm effectiveness (Table S3). Supernatants of the probiotics grown in the presence of the honey expressed a different behavior too. *P. aeruginosa* was the most sensitive pathogenic strain to the action of all *L. casei* supernatants, with inhibition ranging from 8.96% (supernatant of the growth made in the presence of rosemary honey) to 41.94% (observed when we tested the supernatant of the growth of this probiotic made in the presence of mint honey). *A. baumannii* (except in the case of the *L. casei* growth performed in the presence of mint honey) and *E. coli* (except in the test with the supernatant of *L. casei* grown in the presence of basil honey) were two sensitive strains too. *L. monocytogenes* and *S. aureus* were the most resistant strains to the action of the probiotics’ supernatants against whose the supernatants of the control resulted even ineffective. The supernatants of *L. gasseri* exhibited a quite different behavior, being utterly ineffective against *A. baumannii* and, in most cases, also against *E. coli* and *P. aeruginosa*. In contrast, they were active enough against *S. aureus* (with percentages of inhibition up to 54.93% and 76.53%, in the test performed with the supernatants arising from the growth made with mint and oregano honey, respectively). In the test performed with the supernatants from the growth of *L. paracasei* with or without the Lamiaceae honey, we observed that the presence of the honey did not always positively affect the inhibitory effectiveness of the probiotic, whereby the control exhibited percentages of inhibitions ranging between 20.26% (against *A. baumannii*) and 75.52% (against *E. coli*), and, except in a few cases, the action of the control was always more effective than that exhibited by the supernatants of the probiotic grown in the presence of the honey. Lastly, the growth of *L. rhamnosus* with or without honey did not positively influence its antibiofilm activity. However, we should underline that the antibiofilm activity of the control was almost always zero and, based on such a result, we recorded good antibiofilm activity exhibited by the supernatants of this strain grown in the presence of mint honey against *E. coli* (23.42%) but mainly against *P. aeruginosa* (42.03%), and in the presence of savory (inhibition = 20.04%) against *P. aeruginosa*. The MTT test (Table S4) evidenced that the action of the *L. casei* and *L. paracasei* supernatants acted mainly on the metabolism of the pathogenic sessile cells. In some cases, for example, when *L. paracasei* was tested against *P. aeruginosa*, the presence of the honey did not modify the already high effectiveness. In other cases, for example, when the supernatants of *L. casei* were tested against *S. aureus*, the presence of honey during the probiotic growth accentuated the action against the metabolism of the sessile cells, so we observed a percentage of inhibition that increased from 23.41% for the control of *L. casei* to 60.61%, 53.87%, and 58.41% when this probiotic grew in the presence of basil, savory, and thyme honey, respectively. Once again, the supernatants of *L. rhamnosus* were less effective in inhibiting the activity of the sessile cell’s metabolism. However, it was enhanced in some cases, mainly against *A. baumannii*. Thus, we could hypothesize that the fermentative process giving rise to the probiotics in the presence of some Lamiaceae honey caused the formation of some molecules with postbiotics activity, capable of increasing the inhibitory values from zero for the control to 41.01% and 60.55% (against *A. baumannii* and *E. coli*, respectively) when we added thyme honey to the growth medium.

### 2.3. Conclusions

In Table 9, we summarize all the results obtained in our study, dividing them into two large blocks: the antimicrobial activity results and the prebiotic potential exhibited by the six types of honey analyzed.

All types of honey showed a good ability to influence the hydrophobicity of pathogenic strains, and rosemary and thyme honey were the most active from this point of view.

Oregano honey, while effective, showed its potency on a specific set of pathogenic strains, affecting only three out of the total. However, it was the most effective in reducing the hemolytic capacity exhibited by these pathogens.

In the antibiofilm activity test, rosemary honey emerged as the most effective, acting against four out of five strains when included at time zero in the culture medium. However, all types of honey demonstrated the ability to inhibit the mature biofilm, with rosemary, mint, and thyme honey being particularly effective against four out of five strains. Notably, savory honey showed a decrease in its inhibitory power, being effective only against two mature biofilms.

Regarding the prebiotic potential exhibited by the five types of honey using four model probiotic strains, it can be said, in summary, that basil honey influenced both the growth and the hydrophobicity of two probiotic strains, while savory, mint, and thyme honey acted positively only on the growth but not on the hydrophobicity of the probiotics. The antibiofilm activity conducted against pathogenic strains using the supernatants of the four probiotic strains grown in the presence of Lamiaceae honey highlighted that mint and thyme honey were especially able to positively influence the biofilm inhibitory capacity of the growth supernatants of the probiotic strains. However, in general, all the supernatants exhibited more significant inhibitory activity on the metabolism of pathogenic cells within the biofilm, compared to the corresponding controls (consisting of the supernatants of probiotics grown in MRS), and a greater inhibitory propensity was highlighted when the growth of LAB was made in the presence of basil, mint, oregano, and thyme honey and, to a slightly lesser extent, also rosemary and savory honey. The antioxidant activity assays highlighted that basil and mint honey were the most effective for all three tests. Oregano and savory honey performed better than the others in the CFS test, while thyme and rosemary honey performed better in the OHRS test. Finally, the data on cytotoxicity indicate that only basil honey seemed the least effective. The other types of honey, albeit to a greater extent (mint, oregano, and rosemary) or less (thyme and savory), still gave good results regarding antiproliferative activity.

The honey of Lamiaceae evaluated in our study thus exhibited substantial inhibitory effects against five of the most dangerous pathogenic bacteria, acting mainly but not only on their mature biofilm, meaning a more complex situation needs to be fought, particularly for particular population segments such as infants and older people. It also inhibited the metabolic changes occurring in the cells that address the biochemical cell pathway toward the increase in virulence, and through the leakage of nucleic acids and proteins/peptides, weakening the pathogenic bacterial cell and hopefully making it less resistant and less dangerous for the body. Some of them could also decrease their hydrophobicity and hemolytic activity. Concurrently, most of them could enhance the functional properties of five commercial probiotics to increase their potential health benefits to the host. Indeed, the fermentation of the honey by the probiotics produced certain metabolites that can be fully defined as “postbiotics” [79,80]. Future work will evaluate the biochemical characterization of the honey and exploit how its biochemistry can influence other biological properties, both the product itself and the probiotics grown in their presence. Studies are also in progress to evaluate the influence that such kinds of honey can exert in vitro to fight some neurodegenerative diseases, which, as known, can also be triggered by the presence of certain pathogenic bacteria and dysbiosis conditions.

## 3. Materials and Methods

The experiments were performed using six types of Lamiaceae honey: basil honey (Biologique’s choice, London, UK), mint honey (Azienda Prunotto, Piemonte, Italy, batch L97), oregano honey (Apicoltura Rossi SAS, Grosseto, Italy, batch L 1803), rosemary honey (“Le Querce” Azeglio, Turin, Italy, batch BQ12321), savory honey (Apicoltura Salera, Pratola Peligna, Italy, batch L27), and thyme honey (“Le Querce”, Azeglio, Turin, Italy).

The bacterial strains *Acinetobacter baumannii* (ATCC 19606), *Escherichia coli* (DSM 8579), *Pseudomonas aeruginosa* (DSM 50071), *Listeria monocytogenes* (ATCC 7644), and *Staphylococcus aureus* subsp. *aureus* Rosebach (ATCC 25923), utilized in the experiments, were cultured in Luria Broth for 18 h at 37 °C or 35 °C and 80 rpm (Corning LSE, Pisa, Italy) (depending on the strain) before the experiments.

### 3.1. Microbial Adhesion to Solvent

We followed the method of Fratianni et al. [27] to evaluate the microbial adhesion to solvent (MAS). Microorganisms were washed with sterile isotonic saline solution (0.9%). Pellets were resuspended in the same solution to ensure an unchanged initial cell concentration. The absorbance of the cell suspension (A0) was measured at λ = 600 nm (Cary50Bio Varian, Palo Alto, CA, USA); an equal volume of xylene was added, and the mixture was thoroughly mixed for 3 min. After 1 h of incubation, we measured the absorbance of the aqueous phase (A1). The adhesion was calculated from three replicates as a percentage decrease in the optical density of the original bacterial suspension, using the formula: % = [(A0 − A1)/A0]·100.

### 3.2. Minimal Inhibitory Concentration (MIC)

The minimal resazurin microtiter-plate assay was applied to evaluate the MIC [29,81] in flat-bottomed 96-well microtiter plates and then incubated at 37 °C for 24 h (*A. baumannii* grew at 35 °C under the same conditions). Sterile DMSO and tetracycline (dissolved in DMSO, 1 mg/mL) represented the negative and positive controls, respectively. Determinations were performed in triplicate, and the results were expressed as the arithmetic mean ± standard deviation.

### 3.3. Hemolytic Activity Exhibited by the Pathogenic Strains in the Presence of the Honey

Hemolytic activity was assessed using liquid assays, modifying the method of Antunes et al. [82] and Wan et al. [83]. Filter-sterilized supernatants from bacterial cultures grown in Luria Broth added with 8 μg/mL of honey for 14 h and normalized to have the same cell density and were mixed to 10% (final concentration) with sheep defibrinated blood, which was previously washed several times with sterile ice-cold phosphate-buffered saline (PBS) at pH 7.4. After 3 h of incubation at 37 °C with soft agitation and intact erythrocyte harvesting (1000× *g* and 4 °C for 20 min), we measured the amount of hemoglobin released in the supernatants by measuring the OD536. The percentage of hemolysis (P) was calculated using the equation:

P = (X − B)/(T − B),

where X represents the OD_536_ of the sample, while B and T represent the baseline (where the culture supernatant was substituted by deionized water) and total hemolysis (adding to the mixture 0.1% Triton-X100).

### 3.4. Antibiofilm Activity Exhibited by the Honey

#### 3.4.1. The Action of Honey on Immature Biofilm

The capacity of the honey to affect the bacterial biofilm formation was assessed in flat-bottomed 96-well microtiter plates (Falcon, VWR International, Milano, Italy), following the protocol described by Fratianni et al. [29]. Ten microliters of the overnight bacterial cultures (adjusted to 0.5 McFarland with fresh culture broth) were added to each well with 10 µg/mL or 20 µg/mL of honey and sterile Luria–Bertani broth (LB, Sigma Aldrich Italia, Milano, Italy) to a final volume of 250 µL. The plates were protected with parafilm tape and incubated for 48 h at 37 °C or 35 °C, depending on the strain. Following the removal of the planktonic cells, sessile cells were gently cleaned twice with sterile phosphate-buffered saline (PBS), which was finally removed. After 10 min, 200 µL of methanol was added to each well for 15 min to allow the fixation of the sessile cells, which was then discarded. The drying of the plates was followed by the addition of 200 µL of 2% *w/v* crystal violet solution to each well. The staining solution was removed after 20 min, and then the plates were softly washed with sterile PBS and left to dry. Then, 200 µL of glacial acetic acid 20% *w/v* was added to allow the release of the bound dye. The absorbance was measured at λ = 540 nm (Cary 50 Bio, Varian). The percent value of adhesion was calculated with respect to the control (represented by the bacterial cells grown without the presence of the samples, of which the inhibition rate was assumed to be 0%). Triplicate tests were performed, and the results were expressed as the mean ± SD.

#### 3.4.2. The Action of the Honey on Mature Biofilm

Ten microliters of the overnight bacterial cultures (adjusted to 0.5 McFarland with fresh Luria Bertani culture broth cultures) were added to flat-bottomed 96-well microtiter plates to ob a final volume of 250 μL/well. Then, microplates were completely covered with parafilm tape to prevent the evaporation of the material present in the wells and were kept at 37 °C (*A. baumannii* was incubated at 35 °C) for 24 h. The planktonic cells were removed. Two concentrations of the honey, 5 μg/mL and 10 μg/mL of each sample and Luria-Bertani broth, were included, to reach the final volume of 250 μL/well. Plates were then incubated for another 24 h. The sequential steps of the experiment, including the calculation of the percent value of inhibition compared with the untreated bacteria, were performed as described above.

#### 3.4.3. The Action of the Honey on the Bacterial Sessile Cells’ Metabolism

The effect of the two concentrations (10 and 20 μg/mL) of honey added at the start of the bacterial growth and after 24 h was also assessed by evaluating their action on the metabolic activity of the bacterial cells. The assessment was performed using the 3-(4,5-dimethylthiazol-2-yl)-2,5-diphenyltetrazolium bromide (MTT) colorimetric method [19,21]. The honey was added at the beginning of the experiment and after 24 h. After 48 h total of incubation, the planktonic cells were discarded and 150 μL of PBS and 30 μL of 0.3% of MTT (Sigma, Milano, Italy) were included, maintaining the microplates at 37 °C or 35 °C, depending on the strain. The MTT solution was removed after 2 h of incubation, and two washing steps were performed with 200 μL of sterile PBS. The addition of 200 μL of DMSO allowed the dissolution of the formazan crystals that were measured at λ = 570 nm (Cary 50 Bio Varian) after 2 h.

### 3.5. Evaluation of Nuclear and Protein Amount in the Culture Supernatants

The supernatant of cell suspensions treated (grown with honey) and untreated (grown with glucose, which we considered the control) was recovered after centrifugation at 8000 rpm at 4 °C for 15 min and transferred into sterile Eppendorf tubes. The content of nucleic acid release was measured at 260 nm (Cary 50 Bio Varian). Concurrently, the amount of released proteins was calculated using absorbance values at 280 nm [84].

### 3.6. Prebiotic Effect of Honey

*Lacticaseibacillus casei* Shirota (LcS), *Lactobacillus gasseri* LG050, *Lacticaseibacillus paracasei* subsp. *paracasei* I 1688, and *Lacticaseibacillus rhamnosus* GG used in our experiments were bought as commercial formulations available from a local pharmacy. As for previous works [27,29], we chose commercial strains because their probiotic properties have already been ascertained, even at a clinical level, as was compulsory for their commercialization as probiotics. The strains were grown at 37 °C for 16–18 h in MRS without glucose (Liofilchem, Roseto degli Abruzzi, Italy), where glucose was substituted by an equal concentration (*w*/*v*) of the honey. The growth was read at λ = 600 nm (Cary 50Bio, Varian, Palo Alto, CA, USA). The effect of the six kinds of honey on the growth of the lactic bacteria was calculated as a percentage with respect to the control when the strains were grown in the presence of glucose.

#### 3.6.1. Probiotic Adhesion to the Solvent

We followed the method of Fratianni et al. to evaluate the probiotic adhesion to solvent (PAS) [27]. Lactic acid bacteria (LAB) were grown at 37 °C for 16–18 h in MRS without glucose (Liofilchem), where glucose was substituted by an equal concentration (*w*/*v*) of the honey. The cells were then washed with sterile isotonic saline (0.9%). Pellets were collected and re-suspended in the same solution so as to have the same initial cell concentration. The absorbance of the cell suspension (A0) was measured at λ = 600 nm (Cary50Bio Varian), an equal volume of xylene was added, and the mixture was thoroughly mixed for 3 min. After 1 h of incubation, we measured the absorbance of the aqueous phase (A1). The adhesion was calculated from three replicates as a percentage decrease in the optical density of the original bacterial suspension using the formula: % = [(A0 − A1)/A0]·100 and compared to the respective controls, that is, to the strains grown in conventional MRS.

#### 3.6.2. Antioxidant Activity of Probiotics Grown in the Presence of Honey

Lactic acid bacteria were grown in a glucose-free modified MRS medium in which the carbohydrate source (glucose) was substituted by an identical amount (*w*/*v*) of each honey. The strains were grown at 37 °C for 18 h. Aliquots of each bacterial culture were moved to 15 mL tubes, centrifuged (3000 rpm, 4 °C, 10 min), washed three times with sterile isotonic saline, and resuspended in sterile isotonic saline solution to ensure the same initial cell concentration (OD 600).

##### Reducing Power Capacity

The reducing power capacity was assessed by following Lin et al. [85]. In this method, Fe^3+^ is transformed into Fe^2+^ in the presence of possible reducing power. The increase in absorbance of the reaction mixture indicates an increase in reducing power. Five hundred microliters of bacteria were mixed with 0.5 mL of phosphate buffer 0.02 M pH 6.6 and 0.5 mL of 1% potassium ferricyanide. The mixture was incubated in a water bath for 20 min at 50 °C. After cooling, we added 0.5 mL of 10% trichloroacetic acid to the mixture, and then we centrifuged the mixture at 3000 rpm for 10 min. One milliliter of the upper phase was mixed with 1.0 mL of FeCl_3_ 0.1%. The absorbance was measured at λ = 700 nm (Cary Bio Varian, Palo Alto, CA, USA). A blank was prepared without adding honey. Cysteine at various concentrations (from 0.01 to 10 mM) represented the standard for the expression of the reducing activity.

##### The Anti-Lipid Peroxidation Activity (ILAP, Inhibition of Linoleic Acid Peroxidation)

The thiobarbituric acid (TBA) method, based on the monitoring of inhibition of linoleic acid peroxidation [86], was the second method we used to evaluate the antioxidative activity of intact LAB cells. The catalysis of the oxidation was carried out using a Fe/H_2_O_2_ system. Phosphate buffer (0.5 mL, 0.2 M, pH 7.4), 0.5 mL of linoleic acid emulsion, 0.2 mL of FeSO_4_ 0.01%, 0.2 mL of H_2_O_2_, and 0.5 mL of intact cells were mixed and incubated at 37 °C. Blank samples included deionized water. After 12 h of incubation, 2 mL of the reaction solution was mixed with 0.2 mL of trichloroacetic acid TCA) 4%, 2 mL of TBA (0.8%), and 0.2 mL of butylated hydroxytoluene (BHT) (0.4%) to stop additional sample peroxidation while processing. The mixture was incubated at 100 ◦C for 30 min and cooled. After centrifugation (12,000 rpm for 5 min), we measured the absorbance at λ = 532 nm (Cary50Bio, Varian, Palo Alto, CA, USA), and we calculated the percentage of inhibition of linoleic acid peroxidation following the equation: % = [(A_532_sample)/(A_532_blank)]·100.

##### Hydroxyl Radical Scavenging Activity

The hydroxyl radical scavenging activity of LAB grown in the presence of the honey was determined following the method of Guo et al. [86]. The hydroxyl radicals were produced by the Fenton reaction occurring between H_2_O_2_ and FeSO_4_. The reaction was performed in 1.0 mL of 5 mM sodium salicylate, 1.0 mL of 5 mM FeSO_4_, 1.0 mL of LAB, and 1.0 mL of 3 mM H_2_O_2_. The incubation of the reaction mixture was performed at 37 °C for 1 h. The absorbance was measured at λ = 510 nm (Cary50Bio, Varian, Palo Alto, CA, USA). Distilled water constituted the control. The percent of the scavenging rate activity was calculated following the formula: % = [1 − (A_510_sample/A_510_control)]·100.

### 3.7. Antibiofilm Activity of the Supernatants of the LAB Grown in the Presence of the Honey

The four LAB strains were grown at 37 °C for 218 h in an MRS medium, in which glucose was substituted by an equal concentration (*w*/*v*) of the honey. Following centrifugation (3000 rpm, 4 °C, 10 min), the supernatant was recovered and filtered (mesh 0.22 μm, Merck Life Science, Milano, Italy) to carry out the antibacterial and antibiofilm tests.

For the biofilm inhibition test, we grew the five pathogenic strains mentioned above in Luria Bertani broth at 37 °C (*A. baumannii* was incubated at 35 °C) for 18 h. Ten microliters of each bacterial culture were added to a multi-well, previously filled with 80 μL/mL of LAB culture supernatant and Luria-Bertani (Merck Life Science, Milano, Italy) broth up to a final volume of 250 μL. After 24 h of incubation, the inhibitory effect on the adhesion process of pathogens was evaluated following the protocol of Fratianni et al. [17], using the previously described crystal violet and MTT test, and it was measured as the percent with respect to the control (untreated pathogenic bacteria) for which an inhibition = 0% was assumed.

### 3.8. In Vitro Cytotoxicity Assay of Probiotics and Honey for MCF-7, and Caco-2 Cells

The cell viabilities of MCF-7 and Caco-2 cells and the cytotoxic properties of probiotics grown on the six types of honey were analyzed using the 3-(4,5-Dimethylthiazol-2-yl)-2,5-Diphenyltetrazolium Bromide (MTT) Assay.

#### 3.8.1. Cell Cultures

MCF-7 and Caco-2 cell lines were obtained from the American Type Culture Collection (ATCC, Rockville, MD, USA). Cells were grown in Dulbecco’s modified eagle’s medium (DMEM) supplemented with 10% of heat-inactivated fetal bovine serum (FBS), penicillin-streptomycin solution, penicillin (0.010 U L^−1^), and streptomycin (10 mg L^−1^). Cultures were incubated at 37 °C with 5% CO_2_. Cell-free supernatants were diluted with the respective medium as 1/2, 1/4, or 1/6. The same dilutions of honey in the medium (without growing bacteria) were used to determine the effects of solely honey on the cells. Cell-free supernatants of lactobacilli grown on MRS with glucose as a carbon source were used as the control. Then, cancer cells seeded in 96-well plates with a density of 15 × 10^3^ cells per well were treated with a cell-free supernatant of bacteria for 24 h.

#### 3.8.2. 3-(4,5-Dimethylthiazol-2-yl)-2,5-diphenyltetrazolium Bromide (MTT) Assay

After 24 h of incubation, cell viability was determined using a colorimetric MTT assay. Cell survival was determined by the addition of 20 μL of MTT (Promega CellTiter 96^®^ AQueous One Solution Cell; Promega, Madison, WI, USA) for 2 h. The color intensity was measured by a microplate reader (Cary 50 MPR; Varian) at 412 nm. Wells containing cells without any treatment were used as positive controls, and the OD value was used to represent 100% cellular viability. The control and samples were assayed in triplicate for each concentration and replicated three times. The absorbance values were converted into percentages of cell viability using the following formula: Cell viability = Abs sample/Abs control × 100.

### 3.9. Statistical Analysis

Data were expressed as the mean ± SD of three experiments and statistically analyzed using a two-way ANOVA followed by Dunnett’s multiple comparison test, using GraphPad Prism 6.0 (GraphPad Software, Inc., San Diego, CA, USA).

## Figures and Tables

**Table 1:** Minimal Inhibitory Concentration of honey (μg/mL).

MIC	*AB*	*EC*	*LM*	*PA*	*SA*
B	38.0 ± 2.0 ^a^	>50 ^b^	>50 ^b^	>50 ^b^	34.0 ± 1.0 ^a^
M	38.0 ± 1.0 ^a^	>50 ^b^	>50 ^b^	>50 ^b^	34.0 ± 1.0 ^a^
O	40.0 ± 3.0 ^a^	>50 ^b^	>50 ^b^	>50 ^b^	34.0 ± 2.0 ^a^
R	36.0 ± 2.0 ^a^	>50 ^b^	>50 ^b^	38.0 ± 2.0 ^a^	36.0 ± 1.0 ^nd^
S	38.0 ± 1.0 ^a^	>50 ^b^	38 ± 2.0 ^nd^	>50 ^b^	36.0 ± 3.0 ^nd^
T	38.0 ± 3.0 ^a^	38.0 ± 2.0 ^a^	>50 ^b^	>50 ^b^	36.0 ± 1.0 ^nd^
C	30.0 ± 1.0	30.0 ± 2.0	30.0 ± 2.0	28.0 ± 2.0	34.0 ± 1.0

Results are the average (±SD) of three independent experiments. *AB = A. baumannii*; *EC = E. coli*; *LM* = *L. monocytogenes*; *PA = P. aeruginosa*; *SA = S. aureus*. Honey: basil (B); mint (M); oregano(O); rosemary (R); savory (S); thyme (T). As control (C), we used tetracycline. ^a^: *p* < 0.5; ^b^: *p* < 0.01; ^nd^: not detectable (ANOVA followed by Dunnett’s multiple comparison test).

**Table 2:** % of hydrophobicity of bacterial cells grown in the presence of Luria Bertani broth plus 8 μg/mL of basil, mint, oregano, savory, and thyme honey.

	*AB*	*EC*	*LM*	*PA*	*SA*
B	8.6 ± 1.1 ^nd^	0.0 ± 0.0 ^a^	0.0 ± 0.0 ^b^	0.0 ± 0.0 ^a^	0.0 ± 0.0 ^a^
M	2.0 ± 0.1 ^a^	0.0 ± 0.0 ^a^	0.0 ± 0.0 ^b^	0.0 ± 0.0 ^a^	0.0 ± 0.0 ^a^
O	4.76± 0.7 ^a^	0.0 ± 0.0 ^a^	8.3 ± 0.2 ^a^	0.0 ± 0.0 ^a^	0.0 ± 0.0 ^a^
R	0.0 ± 0.0 ^a^	0.0 ± 0.0 ^a^	0.0 ± 0.0 ^b^	0.0 ± 0.0 ^a^	6.0 ± 0.5 ^a^
S	4.7 ± 0.1 ^a^	0.0 ± 0.0 ^a^	0.0 ± 0.0 ^b^	0.0 ± 0.0 ^a^	0.0 ± 0.0 ^a^
T	0.0 ± 0.0 ^a^	0.0 ± 0.0 ^a^	0.0 ± 0.0 ^b^	0.0 ± 0.0 ^a^	0.0 ± 0.0 ^a^
C	8.2 ± 0.6	16.7 ± 1.7	19.7 ± 1.6	11.8 ± 1.3	14.7 ± 1.1

Data are the average (±SD) of three independent experiments. *AB = A. baumannii*; *EC = E. coli*; *LM = L. monocytogenes*; *PA = P. aeruginosa*; *SA = S. aureus.* Honey: basil (B); mint (M); oregano (O); rosemary (R); savory (S); thyme (T). The control (C) is represented by cells grown in Luria Bertani. ^a^: *p* < 0.5; ^b^: *p* < 0.01; ^nd^: not detectable (ANOVA followed by Dunnett’s multiple comparison test).

**Table 3:** Hemolytic activity (%) of bacteria when grown in the presence of 8 μg/mL of honey.

	*AB*	*EC*	*LM*	*PA*	*SA*
B	29.3 ± 1.4 ^b^	7.0 ± 1.0 ^a^	15.3 ± 1.1 ^a^	23.4 ± 2.0 ^nd^	3.1 ± 0.1 ^nd^
M	38.3 ± 2.4 ^a^	2.8 ± 0.2 ^a^	4.5 ± 0.4 ^nd^	20.0 ± 1.6 ^nd^	7.0 ± 1.0 ^nd^
O	37.6 ± 2.2 ^a^	7.9 ± 1.0 ^a^	1.2 ± 0.1 ^nd^	10.4 ± 0.6 ^a^	4.5 ± 0.3 ^nd^
R	40.1 ± 2.3 ^nd^	8.1 ± 0.6 ^a^	2.9 ± 0.3 ^nd^	19.6 ± 1.7 ^nd^	17.7 ± 1.6 ^a^
S	32.5 ± 2.4 ^a^	10.2 ± 1.0 ^nd^	1.9 ± 0.2 ^nd^	22.5 ± 1.7 ^nd^	5.1 ± 1.5 ^nd^
T	44.6 ± 3.3 ^nd^	12.9 ± 1.1 ^nd^	1.5 ± 0.2 ^nd^	9.0 ± 0.6 ^a^	5.1 ± 0.1 ^nd^
C	42.5 ± 3.6	11.6 ± 1.1	3.7 ± 0.5	23.6 ± 1.6	7.2 ± 0.5

Data are the average (±SD) of three independent experiments. *AB = A. baumannii*; *EC = E. coli*; *LM = L. monocytogenes*; *PA = P. aeruginosa*; *SA = S. aureus.* Honey: basil (B); mint (M); oregano(O); rosemary (R); savory (S); thyme (T). The control (C) is represented by cells grown in Luria Bertani. ^a^: *p* < 0.5; ^b^: *p* < 0.01; ^nd^: not detectable (ANOVA followed by Dunnett’s multiple comparison test).

**Table 4:** The amount of nucleic acid in the culture medium after the inclusion of the honey at time zero (0) or after 24 h of growth (24).

	T	*AB*	*EC*	*LM*	*PA*	*SA*
B	0	0.00 ± 0.00 ^a^	0.048 ± 0.00 ^b^	0.00 ± 0.00 ^b^	0.00 ± 0.00 ^b^	0.00 ± 0.00 ^a^
M	0	0.00 ± 0.00 ^a^	0.00 ± 0.00 ^b^	0.00 ± 0.00 ^b^	0.35 ± 0.04 ^b^	0.00 ± 0.00 ^a^
O	0	0.58 ± 0.02 ^a^	0.06 ± 0.0 ^b^	0.02 ± 0.001 ^b^	0.21 ± 0.04 ^b^	0.35 ± 0.06 ^a^
R	0	0.011 ± 0.03 ^a^	0.39 ± 0.04 ^b^	0.78 ± 0.07 ^b^	0.70 ± 0.08 ^b^	0.11 ± 0.05 ^a^
S	0	0.00 ± 0.00 ^a^	0.00 ± 0.00 ^b^	0.00 ± 0.00 ^b^	0.58 ± 0.03 ^b^	0.00 ± 0.00 ^a^
T	0	0.12 ± 0.02 ^a^	0.07 ± 0.01 ^b^	0.85 ± 0.06 ^b^	0.61 ± 0.05 ^b^	0.00 ± 0.00 ^a^
B	24	1.05 ± 0.32 ^a^	0.085 ± 0.00 ^b^	0.00 ± 0.00 ^b^	0.54 ± 0.04 ^b^	0.43 ± 0.1 ^a^
M	24	0.00 ± 0.00 ^a^	0.00 ± 0.00 ^b^	0.60 ± 0.1 ^b^	0.00 ± 0.00 ^b^	0.51 ± 0.12 ^nd^
O	24	1.24 ± 0.12 ^nd^	4.98 ± 0.42 ^b^	5.02 ± 0.32 ^b^	0.56 ± 0.04 ^b^	0.55 ± 0.12 ^nd^
R	24	1.35 ± 0.53 ^nd^	3.89 ± 0.44 ^a^	4.99 ± 0.17 ^b^	0.62 ± 0.06 ^b^	0.11 ± 0.03 ^a^
S	24	0.09 ± 0.02 ^a^	3.88 ± 0.56 ^a^	5.08 ± 0.23 ^b^	0.59 ± 0.04 ^b^	0.13 ± 0.02 ^a^
T	24	0.59 ± 0.12 ^a^	0.00 ± 0.00 ^b^	0.00 ± 0.00 ^b^	0.00 ± 0.00 ^b^	0.00 ± 0.00 ^a^
C		1.18 ± 0.00	2.38 ± 0.00	2.23 ± 0.02	2.75 ± 0.01	0.59 ± 0.003

Each result represents the mean ± SD of three independent experiments, *p* < 0.01 vs. the control group. *AB = A. baumannii*; *EC = E. coli*; *LM = L. monocytogenes*; *PA = P. aeruginosa*; *SA = S. aureus.* Honey: basil (B); mint (M); oregano (O); rosemary (R); savory (S); thyme (T). (C, untreated cells). ^a^: *p* < 0.5; ^b^: *p* < 0.01; ^nd^: not detectable (ANOVA followed by Dunnett’s multiple comparison test).

**Table 5:** The amount of proteic material in the culture medium after the inclusion of the honey at time zero (0) or after 24 h of growth (24).

	T	*AB*	*EC*	*LM*	*PA*	*SA*
B	0	1.60 ± 0.23 ^a^	0.048 ± 0.03 ^nd^	0.00 ± 0.00 ^a^	7.50 ± 0.66 ^c^	0.00 ± 0.00 ^a^
M	0	0.49 ± 0.00 ^a^	8.17 ± 0.80 ^d^	7.57 ± 0.00 ^c^	0.03 ± 0.01 ^a^	0.07 ± 0.02 ^a^
O	0	0.54 ± 0.11 ^a^	0.00 ± 0.00 ^a^	0.00 ± 0.00 ^a^	0.03 ± 0.00 ^a^	0.00 ± 0.00 ^a^
R	0	7.39 ± 0.31 ^c^	0.65 ± 0.21 ^a^	2.19 ± 0.17 ^b^	0.36 ± 0.06 ^a^	0.00 ± 0.00 ^a^
S	0	0.68 ± 0.22 ^a^	0.60 ± 0.14 ^a^	0.09 ± 0.01 ^a^	0.58 ± 0.11 ^a^	0.06 ± 0.02 ^a^
T	0	0.00 ± 0.00 ^a^	0.00 ± 0.00 ^a^	0.00 ± 0.00 ^a^	1.15 ± 0.22 ^b^	0.07 ± 0.02 ^a^
B	24	0.00 ± 0.00 ^a^	0.12 ± 0.01 ^a^	0.092 ± 0.05 ^a^	0.00 ± 0.00 ^a^	0.00 ± 0.00 ^a^
M	24	0.66 ± 0.06 ^a^	0.03 ± 0.00 ^a^	0.00 ± 0.00 ^a^	0.00 ± 0.00 ^a^	0.00 ± 0.00 ^a^
O	24	0.00 ± 0.00 ^a^	0.00 ± 0.00 ^a^	0.00 ± 0.00 ^a^	0.00 ± 0.00 ^a^	0.45 ± 0.03 ^a^
R	24	0.21 ± 0.02 ^a^	0.12 ± 0.02 ^a^	0.00 ± 0.00 ^a^	0.00 ± 0.00 ^a^	0.00 ± 0.00 ^a^
S	24	0.00 ± 0.00 ^a^	0.00 ± 0.00 ^a^	0.00 ± 0.00 ^a^	0.00 ± 0.00 ^a^	0.00 ± 0.00 ^a^
T	24	0.00 ± 0.00 ^a^	0.00 ± 0.00 ^a^	0.00 ± 0.00 ^a^	0.00 ± 0.00 ^a^	0.00 ± 0.00 ^a^
C		1.02 ± 0.00	0.24 ± 0.02	0.28 ± 0.00	0.15 ± 0.00	1.76 ± 0.17

Each result represents the mean ± SD of three independent experiments. *AB = A. baumannii*; *EC = E. coli*; *LM: L. monocytogenes*; *PA = P. aeruginosa*; *SA = S. aureus.* Honey: basil (B); mint (M); oregano (O); rosemary (R); savory (S); thyme (T); control (C, untreated cells). ^a^: *p* < 0.5; ^b^: *p* < 0.01; ^c^: *p* < 0.001; ^d^: *p* < 0.0001; ^nd^: not detectable (ANOVA followed by Dunnett’s multiple comparison test).

**Table 6:** Growth of probiotics with or without(control) the presence of the honey. The data are expressed in terms of λ = 600 nm and are reported as the average (±SD) of three independent experiments. *LC = L. casei Shirota*; *LG = L. gasseri*; *LPC = L. paracasei*; *LR = L. rhamnosus.* Honey: basil (B); mint (M); oregano (O); rosemary (R); savory (S); thyme (T). C represents the growth in conventional MRS containing glucose. a: *p* < 0.5; nd: not detectable (ANOVA followed by Dunnett’s multiple comparison test).

	*LC*	*LG*	*LPC*	*LR*
B	0.833 ± 0.15 ^a^	0.904 ± 0.12 ^nd^	0.722 ± 0.12 ^a^	0.822 ± 0.09 ^a^
M	0.783 ± 0.08 ^nd^	0.887 ± 0.06 ^a^	0.744 ± 0.06 ^nd^	0.821 ± 0.07 ^a^
O	0.91 ± 0.10 ^a^	0.925 ± 0.11 ^nd^	0.733 ± 0.057 ^a^	0.776 ± 0.07 ^nd^
R	0.965 ± 0.07 ^a^	0.971 ± 0.02 ^a^	0.725 ± 0.015 ^a^	0.816 ± 0.12 ^a^
S	0.907 ± 0.06 ^a^	0.974 ± 0.06 ^a^	0.736 ± 0.022 ^a^	0.831 ± 0.02 ^a^
T	0.883 ± 0.01 ^a^	0.907 ± 0.02 ^nd^	0.742 ± 0.017 ^nd^	0.835 ± 0.01 ^a^
C	0.765 ± 0.02	0.927 ± 0.02	0.781 ± 0.05	0.749 ± 0.04

**Table 7:** Hydrophobicity of probiotics evaluated after 3 h of contact with the organic solvent xylene.

	*LC*	*LG*	*LPC*	*LR*
B	0.00 ± 0.00 ^a^	0.00 ± 0.00 ^a^	13.42 ± 0.22 ^b^	1.3 ± 0.05 ^a^
M	0.00 ± 0.00 ^a^	0.00 ± 0.00 ^a^	0.00 ± 0.00 ^a^	0.00 ± 0.00 ^a^
O	0.00 ± 0.00 ^a^	27.26 ± 1.14 ^a^	0.00 ± 0.00 ^a^	0.00 ± 0.00 ^a^
R	9.28 ± 0.11^b^	0.00 ± 0.00 ^a^	0.00 ± 0.00 ^a^	0.00 ± 0.00 ^a^
S	0.00 ± 0.00 ^a^	1.78 ± 0.12 ^a^	0.00 ± 0.00 ^a^	0.00 ± 0.00 ^a^
T	2.41 ± 0.01^a^	0.00 ± 0.00 ^a^	0.00 ± 0.00 ^a^	0.00 ± 0.00 ^a^
C	0.00 ± 0.00 ^a^	0.00 ± 0.00 ^a^	0.00 ± 0.00 ^a^	0.00 ± 0.00 ^a^

The data are expressed in terms of % and are reported as the average (±SD) of three independent experiments. *LC = L. casei Shirota*; *LG = L. gasseri*; *LPC = L. paracasei*; *LR = L. rhamnosus.* Honey: basil (B); mint (M); oregano (O); rosemary (R); savory (S); thyme (T). C represents the growth in conventional MRS containing glucose. ^a^: *p* < 0.5; ^b^: *p* < 0.01 (ANOVA followed by Dunnett’s multiple comparison test).

**Table 8:** Antioxidant activity, evaluated in three ways, exhibited by the LAB grown in the presence of honey.

		*LC*	*LG*	*LPC*	*LR*
ILAP (%)	B	7.6 ± 1.6 ^a^	17.4 ± 1.0 ^a^	18.3 ± 1.7 ^a^	11.7 ± 1.2 ^b^
	M	7.5 ± 0.9 ^a^	17.0 ± 1.8 ^a^	15.9 ± 1.6 ^a^	8.8 ± 1.2 ^b^
	O	5.0 ± 0.6 ^a^	0.0 ± 0.0 ^b^	15.7 ± 2.4 ^a^	6.6 ± 1.5 ^a^
	R	9.2 ± 0.9 ^a^	4.5 ± 0.6 ^a^	0.0 ± 0.0 ^b^	12.4 ± 2.1 ^b^
	S	10.3 ± 1.7 ^a^	8.3 ± 2.2 ^a^	0.0 ± 0.0 ^b^	3.9 ± 0.9 ^a^
	T	5.3 ± 0.7 ^a^	9.7 ± 0.5 ^a^	3.2 ± 1.3 ^a^	0.4 ± 0.1 ^a^
	C	15.8 ± 1.4	11.8 ± 0.3	11.9 ± 0.5	1.30 ± 0.1
OHRS (%)	B	46.1 ± 1.6 ^a^	61.3 ± 2.1 ^c^	37.9 ± 0.4 ^b^	57.5 ± 0.6 ^c^
	M	38.0 ± 1.0 ^nd^	42.9 ± 1.4 ^b^	49.3 ± 0.8 ^c^	39.5 ± 0.0 ^b^
	O	27.4 ± 0.3 ^a^	24.5 ± 0.9 ^a^	24.7 ± 0.4 ^a^	30.7 ± 1.8 ^a^
	R	31.7 ± 1.8 ^a^	40.2 ± 0.2 ^a^	53.3 ± 0.4 ^c^	47.0 ± 8.0 ^b^
	S	30.4 ± 0.9 ^a^	25.7 ± 6.1 ^a^	30.6 ± 0.2 ^a^	39.4 ± 1.1 ^b^
	T	38.7 ± 4.6 ^nd^	59.0 ± 0.5 ^c^	62.9 ± 1.4 ^c^	65.8 ± 1.1 ^c^
	C	36.0 ± 2.8	33.0 ± 2.2	27.4 ± 0.0	26.2 ± 0.6
CFS (mM CystEq)	B	0.86 ± 0.06 ^a^	2.06 ± 0.06 ^a^	2.11 ± 0.1 ^a^	2.01 ± 0.04 ^a^
	M	0.83 ± 0.13 ^a^	1.99 ± 0.06 ^a^	1.17 ± 0.05 ^a^	2.67 ± 0.21 ^b^
	O	1.77 ± 0.15 ^nd^	1.28 ± 0.03 ^a^	1.47 ± 0.1 ^a^	2.27 ± 0.11 ^b^
	R	1.43 ± 0.05 ^nd^	2.22 ± 0.71 ^a^	0.22 ± 0.11 ^a^	3.20 ± 0.09 ^c^
	S	1.63 ± 0.15 ^nd^	2.62 ± 0.17 ^nd^	1.31 ± 0.11 ^nd^	2.8 ± 0.11 ^c^
	T	1.23 ± 0.02 ^nd^	1.63 ± 0.02 ^a^	0.25 ± 0.03 ^a^	1.22 ± 0.02 ^a^
	C	1.58 ± 0.09	2.58 ± 0.11	1.38 ± 0.06	1.02 ± 0.06

The data are reported as the average (±SD) of three independent experiments. ILAP: Inhibition of Linoleic Acid Peroxidation; OHRS: Hydroxyl Radical Scavenging Activity; CFS: cell-free supernatant activity. *LC = L. casei Shirota*; *LG = L. gasseri*; *LPC = L. paracasei*; *LR = L. rhamnosus.* Honey: basil (B); mint (M); oregano(O); rosemary (R); savory (S); thyme (T). C represents the growth in conventional MRS containing glucose. ^a^: *p* < 0.5; ^b^: *p* < 0.01; ^c^: *p* < 0.001; ^nd^: not detectable (ANOVA followed by Dunnett’s multiple comparison test).

**Table 9:** Summary of the effectiveness of basil mint, oregano, rosemary, savory, and thyme honey as antimicrobial and prebiotic agents. The number of “+” is commensurate with the behavior of the honey, and indicates the major or minor effectiveness of the kinds of honey depending on the tests performed. The “−” sign indicates no effectiveness.

	Antimicrobial Activity							
	hydrophobicity	hemolytic activity	CV0	MTT 0	CV 24	MTT24		
basil	++++	+++	++	++	++++	−		
mint	++++	++++	++	++	+++++	−		
oregano	+++	+++++	++	++	++++	−		
rosemary	+++++	++++	++++	++	+++++	−		
savory	++++	++++	+++	++	++	−		
thyme	+++++	+++	++	++	+++++	−		
	probiotic activity							
	hydrophobicity	growth	CV0	MTT 0	antioxidant ILAP	antioxidant OHRS	antioxidant CFS	cytotoxic activity
basil	++	++	+	+++++	+++	+++++	++	−
mint	-	++	+++++	+++++	+++	+++	+	++++
oregano	+	+	++	+++++	++	−	+++	++++
rosemary	+	−	+++	++++	+	+++	+	++++
savory	-	++	++	+++	+	++	+++	+
thyme	+	++	+++++	+++++	−	++++	+	++

## Data Availability

The data presented in this study are available upon request from the corresponding author.

## Supplementary Materials

The following supporting information can be downloaded at: https://www.mdpi.com/article/10.3390/antibiotics13090868/s1, Table S1. Biofilm inhibitory activity (expressed as percentage) of the six kinds of Lamiaceae honey, basil (B), mint (M), oregano (O), rosemary (R), savory (S), and thyme (T) added at time zero and after 24 hours of incubation against the pathogens Acinetobacter baumannii (AB), Escherichia coli (EC), Listeria monocytogenes (LM), Pseudomonas aeruginosa (PA), and Staphylococcus aureus (SA). Table S2. Inhibitory activity (expressed as percentage) of the six kinds of Lamiaceae honey, basil (B), mint (M), oregano (O), rosemary (R), savory (S), and thyme (T) added at time zero and after 24 hours of incubation against the metabolism of sessile cells of pathogens Acinetobacter baumannii (AB), Escherichia coli (EC), Listeria monocytogenes (LM), Pseudomonas aeruginosa (PA), and Staphylococcus aureus (SA). Table S3. Inhibitory activity of L. casei Shirota, L. gasseri, L. paracasei, L. rhamnosus supernatant against the biofilm of A. baumannii (AB), E. coli (EC), L. monocytogenes (LM), P. aeruginosa (PA), and S. aureus (SA). The probiotic strain was grown with or without the presence of the six types of honey. Table S4. Inhibitory activity of L. casei Shirota, L. gasseri, L. paracasei, L. rhamnosus supernatant against the metabolism of sessile cells of A. baumannii (AB), E. coli (EC), L. monocytogenes (LM), P. aeruginosa (PA), and S. aureus (SA). The probiotic strain was grown with or without the presence of the six types of honey. Figure S1. The in vitro anti-proliferative effects of L. casei versus MCF-7 and Caco-2 cell lines (a), and of L. gasseri against MCF-7 and Caco-2 cells treated with different dilutions of Lactobacillus-free surnatants (b). Figure S2. The in vitro anti-proliferative activities of L. paracasei against MCF-7 and Caco-2 cell lines (a), and of L. rhamnosus on MCF-7 and Caco-2 cells treated with various dilutions of lactobacillus-free supernatants (b).
