# Supplementary material for: Antibacterial Activity and Prebiotic Properties of Six Types of Lamiaceae Honey
Source: Antibiotics (Basel). 2024 Sep 10;13(9):868. doi: 10.3390/antibiotics13090868 (PMC11428214; doi:10.3390/antibiotics13090868)
Supplement: Supplementary file 1 [file antibiotics-13-00868-s001.zip › antibiotics-3111164-supplementary.pptx]

## Slide 1
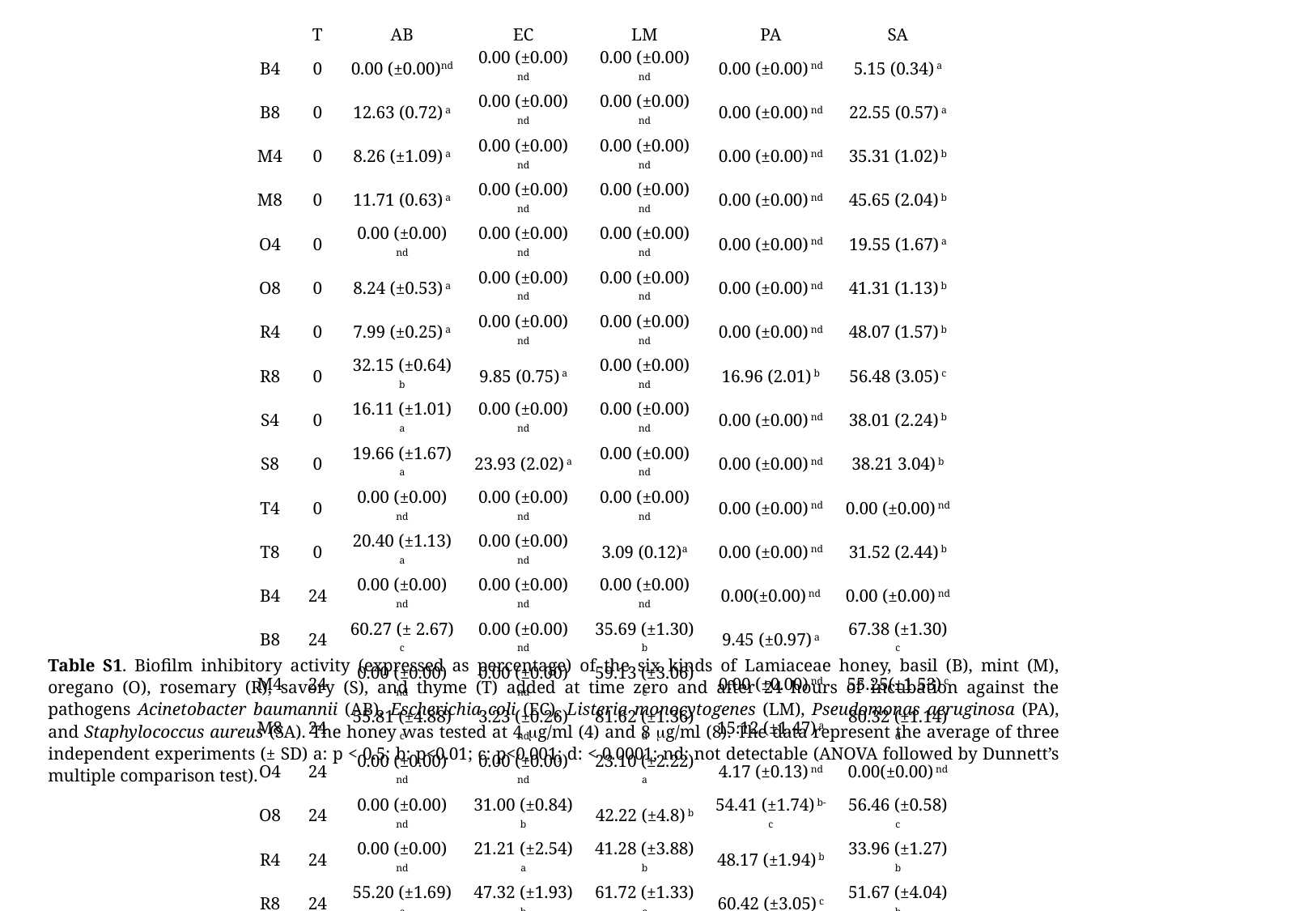

| | T | AB | EC | LM | PA | SA |
| --- | --- | --- | --- | --- | --- | --- |
| B4 | 0 | 0.00 (±0.00)nd | 0.00 (±0.00) nd | 0.00 (±0.00) nd | 0.00 (±0.00) nd | 5.15 (0.34) a |
| B8 | 0 | 12.63 (0.72) a | 0.00 (±0.00) nd | 0.00 (±0.00) nd | 0.00 (±0.00) nd | 22.55 (0.57) a |
| M4 | 0 | 8.26 (±1.09) a | 0.00 (±0.00) nd | 0.00 (±0.00) nd | 0.00 (±0.00) nd | 35.31 (1.02) b |
| M8 | 0 | 11.71 (0.63) a | 0.00 (±0.00) nd | 0.00 (±0.00) nd | 0.00 (±0.00) nd | 45.65 (2.04) b |
| O4 | 0 | 0.00 (±0.00) nd | 0.00 (±0.00) nd | 0.00 (±0.00) nd | 0.00 (±0.00) nd | 19.55 (1.67) a |
| O8 | 0 | 8.24 (±0.53) a | 0.00 (±0.00) nd | 0.00 (±0.00) nd | 0.00 (±0.00) nd | 41.31 (1.13) b |
| R4 | 0 | 7.99 (±0.25) a | 0.00 (±0.00) nd | 0.00 (±0.00) nd | 0.00 (±0.00) nd | 48.07 (1.57) b |
| R8 | 0 | 32.15 (±0.64) b | 9.85 (0.75) a | 0.00 (±0.00) nd | 16.96 (2.01) b | 56.48 (3.05) c |
| S4 | 0 | 16.11 (±1.01) a | 0.00 (±0.00) nd | 0.00 (±0.00) nd | 0.00 (±0.00) nd | 38.01 (2.24) b |
| S8 | 0 | 19.66 (±1.67) a | 23.93 (2.02) a | 0.00 (±0.00) nd | 0.00 (±0.00) nd | 38.21 3.04) b |
| T4 | 0 | 0.00 (±0.00) nd | 0.00 (±0.00) nd | 0.00 (±0.00) nd | 0.00 (±0.00) nd | 0.00 (±0.00) nd |
| T8 | 0 | 20.40 (±1.13) a | 0.00 (±0.00) nd | 3.09 (0.12)a | 0.00 (±0.00) nd | 31.52 (2.44) b |
| B4 | 24 | 0.00 (±0.00) nd | 0.00 (±0.00) nd | 0.00 (±0.00) nd | 0.00(±0.00) nd | 0.00 (±0.00) nd |
| B8 | 24 | 60.27 (± 2.67) c | 0.00 (±0.00) nd | 35.69 (±1.30) b | 9.45 (±0.97) a | 67.38 (±1.30) c |
| M4 | 24 | 0.00 (±0.00) nd | 0.00 (±0.00) nd | 59.13 (±3.06) c | 0.00 (±0.00) nd | 55.25(±1.53) c |
| M8 | 24 | 55.81 (±4.88) c | 3.23 (±0.26) nd | 81.62 (±1.36) d | 15.12 (±1.47) a | 80.32 (±1.14) d |
| O4 | 24 | 0.00 (±0.00) nd | 0.00 (±0.00) nd | 23.10 (±2.22) a | 4.17 (±0.13) nd | 0.00(±0.00) nd |
| O8 | 24 | 0.00 (±0.00) nd | 31.00 (±0.84) b | 42.22 (±4.8) b | 54.41 (±1.74) b-c | 56.46 (±0.58) c |
| R4 | 24 | 0.00 (±0.00) nd | 21.21 (±2.54) a | 41.28 (±3.88) b | 48.17 (±1.94) b | 33.96 (±1.27) b |
| R8 | 24 | 55.20 (±1.69) c | 47.32 (±1.93) b | 61.72 (±1.33) c | 60.42 (±3.05) c | 51.67 (±4.04) b |
| S4 | 24 | 0.00 (±0.00) nd | 60.05 (±2.54) c | 12.17 (±1.04) a | 0.00 (±0.00) nd | 49.67 (±3.13) b |
| S8 | 24 | 0.00 (±0.00) nd | 63.95 (±1.11) c | 70.94 (±0.97) c | 0.00 (±0.00) nd | 0.00 (±0.00) nd |
| T4 | 24 | 0.00 (±0.00) nd | 0.00 (±0.00) nd | 0.00 (±0.00) nd | 0.00 (±0.00) nd | 43.84 (±3.32) |
| T8 | 24 | 50.11 (±1.67) b | 50.01 (±2.14) b | 68.85 (±3.01) c | 21.83 (±1.57) a | 53.54 (±2.67) b |
Table S1. Biofilm inhibitory activity (expressed as percentage) of the six kinds of Lamiaceae honey, basil (B), mint (M), oregano (O), rosemary (R), savory (S), and thyme (T) added at time zero and after 24 hours of incubation against the pathogens Acinetobacter baumannii (AB), Escherichia coli (EC), Listeria monocytogenes (LM), Pseudomonas aeruginosa (PA), and Staphylococcus aureus (SA). The honey was tested at 4 mg/ml (4) and 8 μg/ml (8). The data represent the average of three independent experiments (± SD) a: p < 0.5; b: p<0.01; c: p<0.001; d: < 0.0001; nd: not detectable (ANOVA followed by Dunnett’s multiple comparison test).

## Slide 2
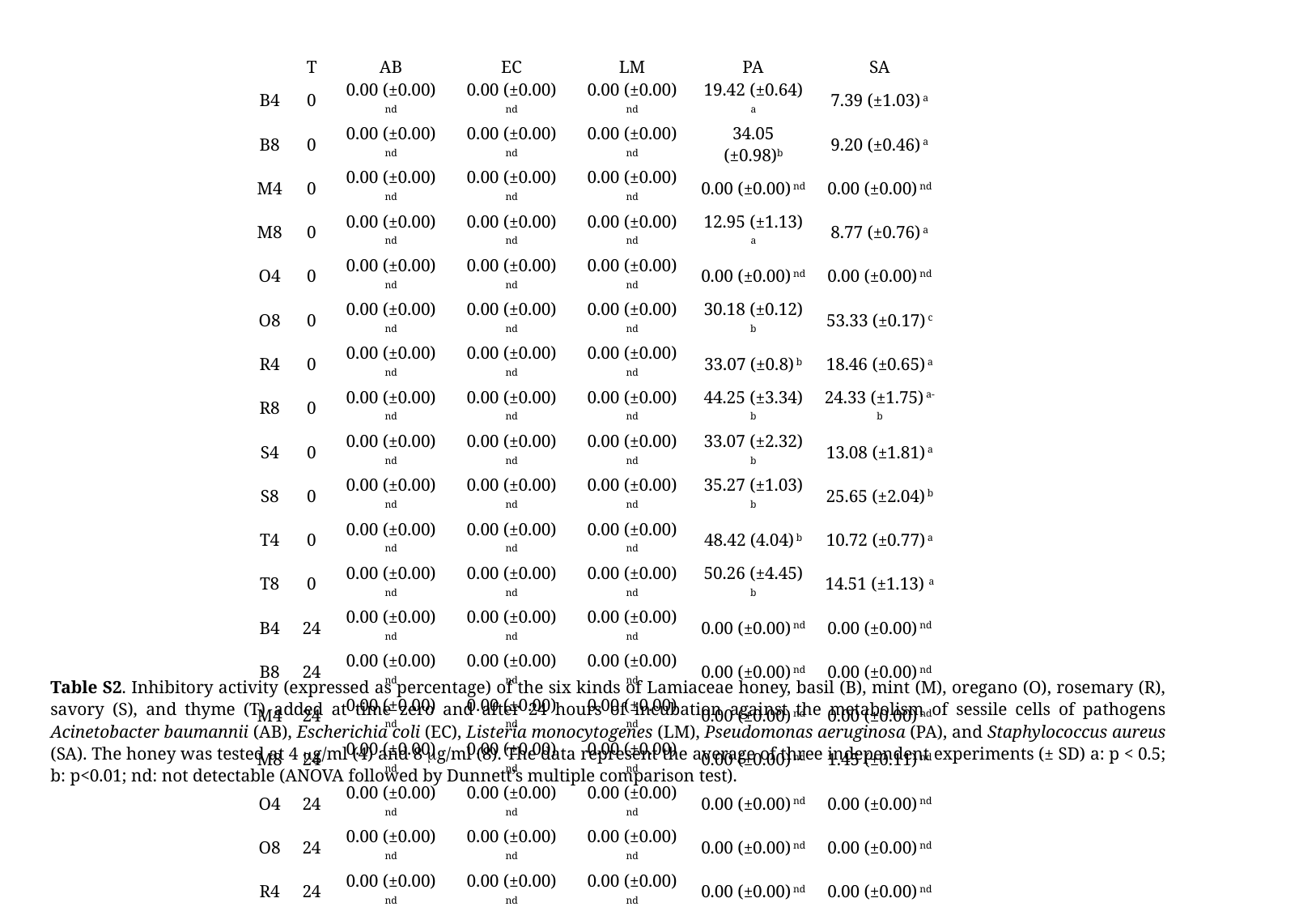

| | T | AB | EC | LM | PA | SA |
| --- | --- | --- | --- | --- | --- | --- |
| B4 | 0 | 0.00 (±0.00) nd | 0.00 (±0.00) nd | 0.00 (±0.00) nd | 19.42 (±0.64) a | 7.39 (±1.03) a |
| B8 | 0 | 0.00 (±0.00) nd | 0.00 (±0.00) nd | 0.00 (±0.00) nd | 34.05 (±0.98)b | 9.20 (±0.46) a |
| M4 | 0 | 0.00 (±0.00) nd | 0.00 (±0.00) nd | 0.00 (±0.00) nd | 0.00 (±0.00) nd | 0.00 (±0.00) nd |
| M8 | 0 | 0.00 (±0.00) nd | 0.00 (±0.00) nd | 0.00 (±0.00) nd | 12.95 (±1.13) a | 8.77 (±0.76) a |
| O4 | 0 | 0.00 (±0.00) nd | 0.00 (±0.00) nd | 0.00 (±0.00) nd | 0.00 (±0.00) nd | 0.00 (±0.00) nd |
| O8 | 0 | 0.00 (±0.00) nd | 0.00 (±0.00) nd | 0.00 (±0.00) nd | 30.18 (±0.12) b | 53.33 (±0.17) c |
| R4 | 0 | 0.00 (±0.00) nd | 0.00 (±0.00) nd | 0.00 (±0.00) nd | 33.07 (±0.8) b | 18.46 (±0.65) a |
| R8 | 0 | 0.00 (±0.00) nd | 0.00 (±0.00) nd | 0.00 (±0.00) nd | 44.25 (±3.34) b | 24.33 (±1.75) a-b |
| S4 | 0 | 0.00 (±0.00) nd | 0.00 (±0.00) nd | 0.00 (±0.00) nd | 33.07 (±2.32) b | 13.08 (±1.81) a |
| S8 | 0 | 0.00 (±0.00) nd | 0.00 (±0.00) nd | 0.00 (±0.00) nd | 35.27 (±1.03) b | 25.65 (±2.04) b |
| T4 | 0 | 0.00 (±0.00) nd | 0.00 (±0.00) nd | 0.00 (±0.00) nd | 48.42 (4.04) b | 10.72 (±0.77) a |
| T8 | 0 | 0.00 (±0.00) nd | 0.00 (±0.00) nd | 0.00 (±0.00) nd | 50.26 (±4.45) b | 14.51 (±1.13) a |
| B4 | 24 | 0.00 (±0.00) nd | 0.00 (±0.00) nd | 0.00 (±0.00) nd | 0.00 (±0.00) nd | 0.00 (±0.00) nd |
| B8 | 24 | 0.00 (±0.00) nd | 0.00 (±0.00) nd | 0.00 (±0.00) nd | 0.00 (±0.00) nd | 0.00 (±0.00) nd |
| M4 | 24 | 0.00 (±0.00) nd | 0.00 (±0.00) nd | 0.00 (±0.00) nd | 0.00 (±0.00) nd | 0.00 (±0.00) nd |
| M8 | 24 | 0.00 (±0.00) nd | 0.00 (±0.00) nd | 0.00 (±0.00) nd | 0.00 (±0.00) nd | 1.45 (±0.11) nd |
| O4 | 24 | 0.00 (±0.00) nd | 0.00 (±0.00) nd | 0.00 (±0.00) nd | 0.00 (±0.00) nd | 0.00 (±0.00) nd |
| O8 | 24 | 0.00 (±0.00) nd | 0.00 (±0.00) nd | 0.00 (±0.00) nd | 0.00 (±0.00) nd | 0.00 (±0.00) nd |
| R4 | 24 | 0.00 (±0.00) nd | 0.00 (±0.00) nd | 0.00 (±0.00) nd | 0.00 (±0.00) nd | 0.00 (±0.00) nd |
| R8 | 24 | 0.00 (±0.00) nd | 0.00 (±0.00) nd | 8.81 (±0.72) a | 0.00 (±0.00) nd | 0.00 (±0.00) nd |
| S4 | 24 | 0.00 (±0.00) nd | 0.00 (±0.00) nd | 0.00 (±0.00) nd | 0.00 (±0.00) nd | 0.00 (±0.00) nd |
| S8 | 24 | 0.00 (±0.00) nd | 0.00 (±0.00) nd | 0.00 (±0.00) nd | 0.00 (±0.00) nd | 0.00 (±0.00) nd |
| T4 | 24 | 0.00 (±0.00) nd | 0.00 (±0.00) nd | 0.96 (±0.15) nd | 0.00 (±0.00) nd | 0.00 (±0.00) nd |
| T8 | 24 | 0.00 (±0.00) nd | 0.00 (±0.00) nd | 27.48 (±2.24) b | 0.00 (±0.00) nd | 0.00 (±0.00) nd |
Table S2. Inhibitory activity (expressed as percentage) of the six kinds of Lamiaceae honey, basil (B), mint (M), oregano (O), rosemary (R), savory (S), and thyme (T) added at time zero and after 24 hours of incubation against the metabolism of sessile cells of pathogens Acinetobacter baumannii (AB), Escherichia coli (EC), Listeria monocytogenes (LM), Pseudomonas aeruginosa (PA), and Staphylococcus aureus (SA). The honey was tested at 4 mg/ml (4) and 8 μg/ml (8). The data represent the average of three independent experiments (± SD) a: p < 0.5; b: p<0.01; nd: not detectable (ANOVA followed by Dunnett’s multiple comparison test).

## Slide 3
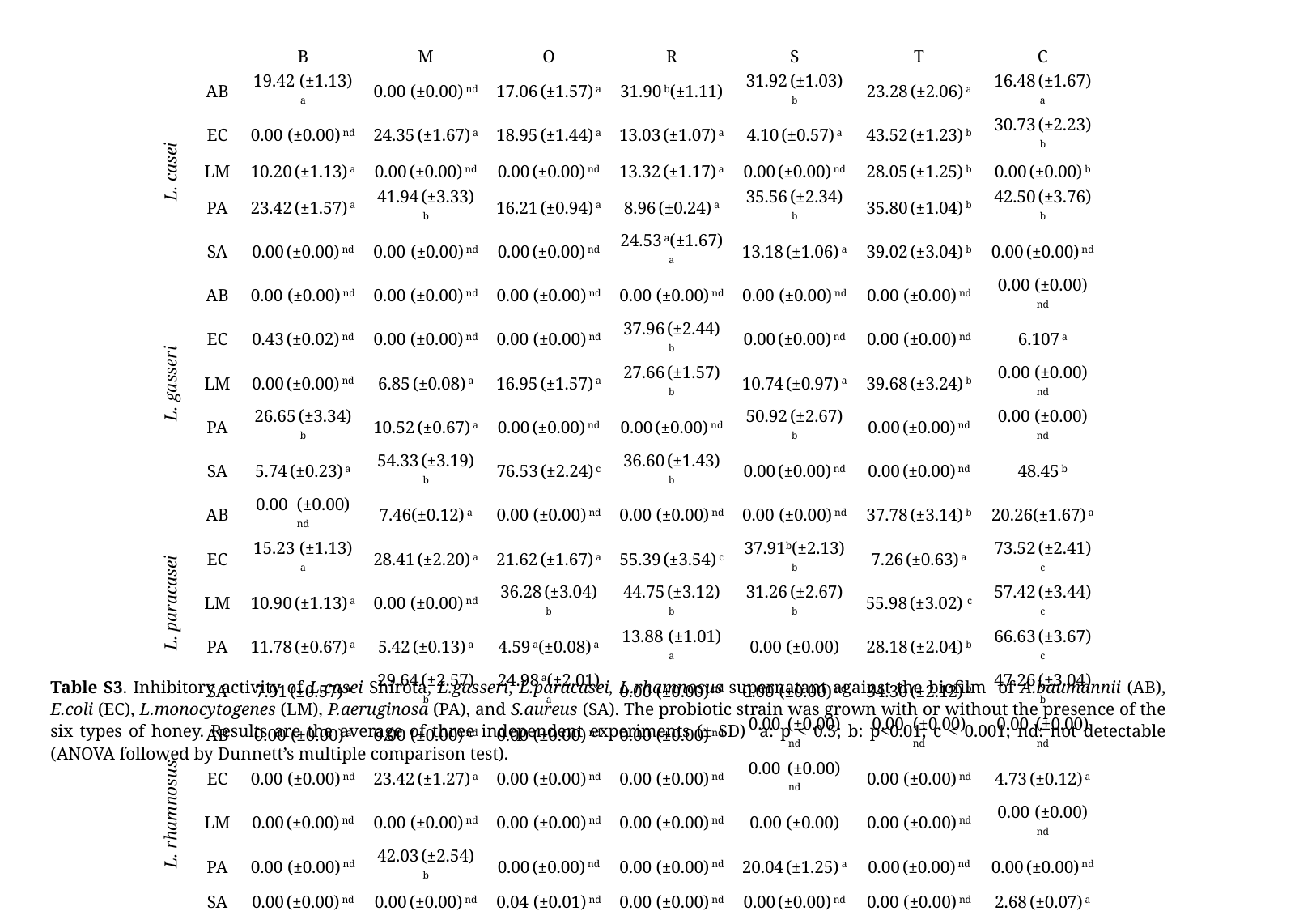

| | | B | M | O | R | S | T | C |
| --- | --- | --- | --- | --- | --- | --- | --- | --- |
| L. casei | AB | 19.42 (±1.13) a | 0.00 (±0.00) nd | 17.06 (±1.57) a | 31.90 b(±1.11) | 31.92 (±1.03) b | 23.28 (±2.06) a | 16.48 (±1.67) a |
| | EC | 0.00 (±0.00) nd | 24.35 (±1.67) a | 18.95 (±1.44) a | 13.03 (±1.07) a | 4.10 (±0.57) a | 43.52 (±1.23) b | 30.73 (±2.23) b |
| | LM | 10.20 (±1.13) a | 0.00 (±0.00) nd | 0.00 (±0.00) nd | 13.32 (±1.17) a | 0.00 (±0.00) nd | 28.05 (±1.25) b | 0.00 (±0.00) b |
| | PA | 23.42 (±1.57) a | 41.94 (±3.33) b | 16.21 (±0.94) a | 8.96 (±0.24) a | 35.56 (±2.34) b | 35.80 (±1.04) b | 42.50 (±3.76) b |
| | SA | 0.00 (±0.00) nd | 0.00 (±0.00) nd | 0.00 (±0.00) nd | 24.53 a(±1.67) a | 13.18 (±1.06) a | 39.02 (±3.04) b | 0.00 (±0.00) nd |
| L. gasseri | AB | 0.00 (±0.00) nd | 0.00 (±0.00) nd | 0.00 (±0.00) nd | 0.00 (±0.00) nd | 0.00 (±0.00) nd | 0.00 (±0.00) nd | 0.00 (±0.00) nd |
| | EC | 0.43 (±0.02) nd | 0.00 (±0.00) nd | 0.00 (±0.00) nd | 37.96 (±2.44) b | 0.00 (±0.00) nd | 0.00 (±0.00) nd | 6.107 a |
| | LM | 0.00 (±0.00) nd | 6.85 (±0.08) a | 16.95 (±1.57) a | 27.66 (±1.57) b | 10.74 (±0.97) a | 39.68 (±3.24) b | 0.00 (±0.00) nd |
| | PA | 26.65 (±3.34) b | 10.52 (±0.67) a | 0.00 (±0.00) nd | 0.00 (±0.00) nd | 50.92 (±2.67) b | 0.00 (±0.00) nd | 0.00 (±0.00) nd |
| | SA | 5.74 (±0.23) a | 54.33 (±3.19) b | 76.53 (±2.24) c | 36.60 (±1.43) b | 0.00 (±0.00) nd | 0.00 (±0.00) nd | 48.45 b |
| L. paracasei | AB | 0.00 (±0.00) nd | 7.46(±0.12) a | 0.00 (±0.00) nd | 0.00 (±0.00) nd | 0.00 (±0.00) nd | 37.78 (±3.14) b | 20.26(±1.67) a |
| | EC | 15.23 (±1.13) a | 28.41 (±2.20) a | 21.62 (±1.67) a | 55.39 (±3.54) c | 37.91b(±2.13) b | 7.26 (±0.63) a | 73.52 (±2.41) c |
| | LM | 10.90 (±1.13) a | 0.00 (±0.00) nd | 36.28 (±3.04) b | 44.75 (±3.12) b | 31.26 (±2.67) b | 55.98 (±3.02) c | 57.42 (±3.44) c |
| | PA | 11.78 (±0.67) a | 5.42 (±0.13) a | 4.59 a(±0.08) a | 13.88 (±1.01) a | 0.00 (±0.00) | 28.18 (±2.04) b | 66.63 (±3.67) c |
| | SA | 7.91 (±0.57) a | 29.64 (±2.57) b | 24.98 a(±2.01) a | 0.00 (±0.00) nd | 0.00 (±0.00) nd | 34.30 (±2.12) b | 47.26 (±3.04) b |
| L. rhamnosus | AB | 0.00 (±0.00) a | 0.00 (±0.00) nd | 0.00 (±0.00) nd | 0.00 (±0.00) nd | 0.00 (±0.00) nd | 0.00 (±0.00) nd | 0.00 (±0.00) nd |
| | EC | 0.00 (±0.00) nd | 23.42 (±1.27) a | 0.00 (±0.00) nd | 0.00 (±0.00) nd | 0.00 (±0.00) nd | 0.00 (±0.00) nd | 4.73 (±0.12) a |
| | LM | 0.00 (±0.00) nd | 0.00 (±0.00) nd | 0.00 (±0.00) nd | 0.00 (±0.00) nd | 0.00 (±0.00) | 0.00 (±0.00) nd | 0.00 (±0.00) nd |
| | PA | 0.00 (±0.00) nd | 42.03 (±2.54) b | 0.00 (±0.00) nd | 0.00 (±0.00) nd | 20.04 (±1.25) a | 0.00 (±0.00) nd | 0.00 (±0.00) nd |
| | SA | 0.00 (±0.00) nd | 0.00 (±0.00) nd | 0.04 (±0.01) nd | 0.00 (±0.00) nd | 0.00 (±0.00) nd | 0.00 (±0.00) nd | 2.68 (±0.07) a |
Table S3. Inhibitory activity of L.casei Shirota, L.gasseri, L.paracasei, L.rhamnosus supernatant against the biofilm of A.baumannii (AB), E.coli (EC), L.monocytogenes (LM), P.aeruginosa (PA), and S.aureus (SA). The probiotic strain was grown with or without the presence of the six types of honey. Results are the average of three independent experiments (± SD) a: p < 0.5; b: p<0.01; c < 0.001; nd: not detectable (ANOVA followed by Dunnett’s multiple comparison test).

## Slide 4
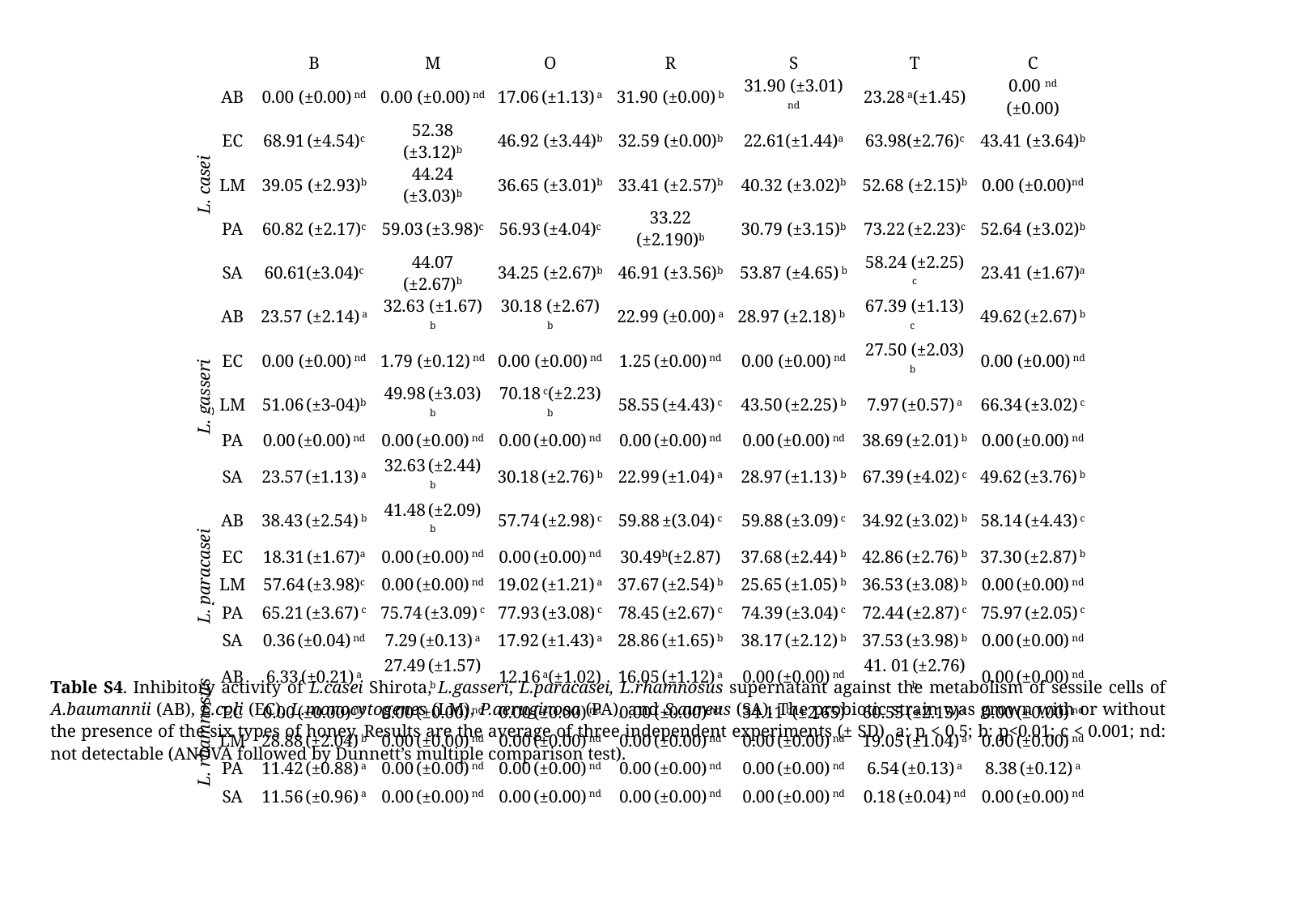

| | | B | M | O | R | S | T | C |
| --- | --- | --- | --- | --- | --- | --- | --- | --- |
| L. casei | AB | 0.00 (±0.00) nd | 0.00 (±0.00) nd | 17.06 (±1.13) a | 31.90 (±0.00) b | 31.90 (±3.01) nd | 23.28 a(±1.45) | 0.00 nd (±0.00) |
| | EC | 68.91 (±4.54)c | 52.38 (±3.12)b | 46.92 (±3.44)b | 32.59 (±0.00)b | 22.61(±1.44)a | 63.98(±2.76)c | 43.41 (±3.64)b |
| | LM | 39.05 (±2.93)b | 44.24 (±3.03)b | 36.65 (±3.01)b | 33.41 (±2.57)b | 40.32 (±3.02)b | 52.68 (±2.15)b | 0.00 (±0.00)nd |
| | PA | 60.82 (±2.17)c | 59.03 (±3.98)c | 56.93 (±4.04)c | 33.22 (±2.190)b | 30.79 (±3.15)b | 73.22 (±2.23)c | 52.64 (±3.02)b |
| | SA | 60.61(±3.04)c | 44.07 (±2.67)b | 34.25 (±2.67)b | 46.91 (±3.56)b | 53.87 (±4.65) b | 58.24 (±2.25) c | 23.41 (±1.67)a |
| L. gasseri | AB | 23.57 (±2.14) a | 32.63 (±1.67) b | 30.18 (±2.67) b | 22.99 (±0.00) a | 28.97 (±2.18) b | 67.39 (±1.13) c | 49.62 (±2.67) b |
| | EC | 0.00 (±0.00) nd | 1.79 (±0.12) nd | 0.00 (±0.00) nd | 1.25 (±0.00) nd | 0.00 (±0.00) nd | 27.50 (±2.03) b | 0.00 (±0.00) nd |
| | LM | 51.06 (±3-04)b | 49.98 (±3.03) b | 70.18 c(±2.23) b | 58.55 (±4.43) c | 43.50 (±2.25) b | 7.97 (±0.57) a | 66.34 (±3.02) c |
| | PA | 0.00 (±0.00) nd | 0.00 (±0.00) nd | 0.00 (±0.00) nd | 0.00 (±0.00) nd | 0.00 (±0.00) nd | 38.69 (±2.01) b | 0.00 (±0.00) nd |
| | SA | 23.57 (±1.13) a | 32.63 (±2.44) b | 30.18 (±2.76) b | 22.99 (±1.04) a | 28.97 (±1.13) b | 67.39 (±4.02) c | 49.62 (±3.76) b |
| L. paracasei | AB | 38.43 (±2.54) b | 41.48 (±2.09) b | 57.74 (±2.98) c | 59.88 ±(3.04) c | 59.88 (±3.09) c | 34.92 (±3.02) b | 58.14 (±4.43) c |
| | EC | 18.31 (±1.67)a | 0.00 (±0.00) nd | 0.00 (±0.00) nd | 30.49b(±2.87) | 37.68 (±2.44) b | 42.86 (±2.76) b | 37.30 (±2.87) b |
| | LM | 57.64 (±3.98)c | 0.00 (±0.00) nd | 19.02 (±1.21) a | 37.67 (±2.54) b | 25.65 (±1.05) b | 36.53 (±3.08) b | 0.00 (±0.00) nd |
| | PA | 65.21 (±3.67) c | 75.74 (±3.09) c | 77.93 (±3.08) c | 78.45 (±2.67) c | 74.39 (±3.04) c | 72.44 (±2.87) c | 75.97 (±2.05) c |
| | SA | 0.36 (±0.04) nd | 7.29 (±0.13) a | 17.92 (±1.43) a | 28.86 (±1.65) b | 38.17 (±2.12) b | 37.53 (±3.98) b | 0.00 (±0.00) nd |
| L. rhamnosus | AB | 6.33 (±0.21) a | 27.49 (±1.57) b | 12.16 a(±1.02) | 16.05 (±1.12) a | 0.00 (±0.00) nd | 41. 01 (±2.76) b | 0.00 (±0.00) nd |
| | EC | 0.00 (±0.00) nd | 0.00 (±0.00) nd | 0.00 (±0.00) nd | 0.00 (±0.00) nd | 34.11 b(±2.65) | 60.55 (±2.15) c | 0.00 (±0.00) nd |
| | LM | 28.88 (±2.04) b | 0.00 (±0.00) nd | 0.00 (±0.00) nd | 0.00 (±0.00) nd | 0.00 (±0.00) nd | 19.05 (±1.04) a | 0.00 (±0.00) nd |
| | PA | 11.42 (±0.88) a | 0.00 (±0.00) nd | 0.00 (±0.00) nd | 0.00 (±0.00) nd | 0.00 (±0.00) nd | 6.54 (±0.13) a | 8.38 (±0.12) a |
| | SA | 11.56 (±0.96) a | 0.00 (±0.00) nd | 0.00 (±0.00) nd | 0.00 (±0.00) nd | 0.00 (±0.00) nd | 0.18 (±0.04) nd | 0.00 (±0.00) nd |
Table S4. Inhibitory activity of L.casei Shirota, L.gasseri, L.paracasei, L.rhamnosus supernatant against the metabolism of sessile cells of A.baumannii (AB), E.coli (EC), L.monocytogenes (LM), P.aeruginosa (PA), and S.aureus (SA). The probiotic strain was grown with or without the presence of the six types of honey. Results are the average of three independent experiments (± SD) a: p < 0.5; b: p<0.01; c < 0.001; nd: not detectable (ANOVA followed by Dunnett’s multiple comparison test).

## Slide 5
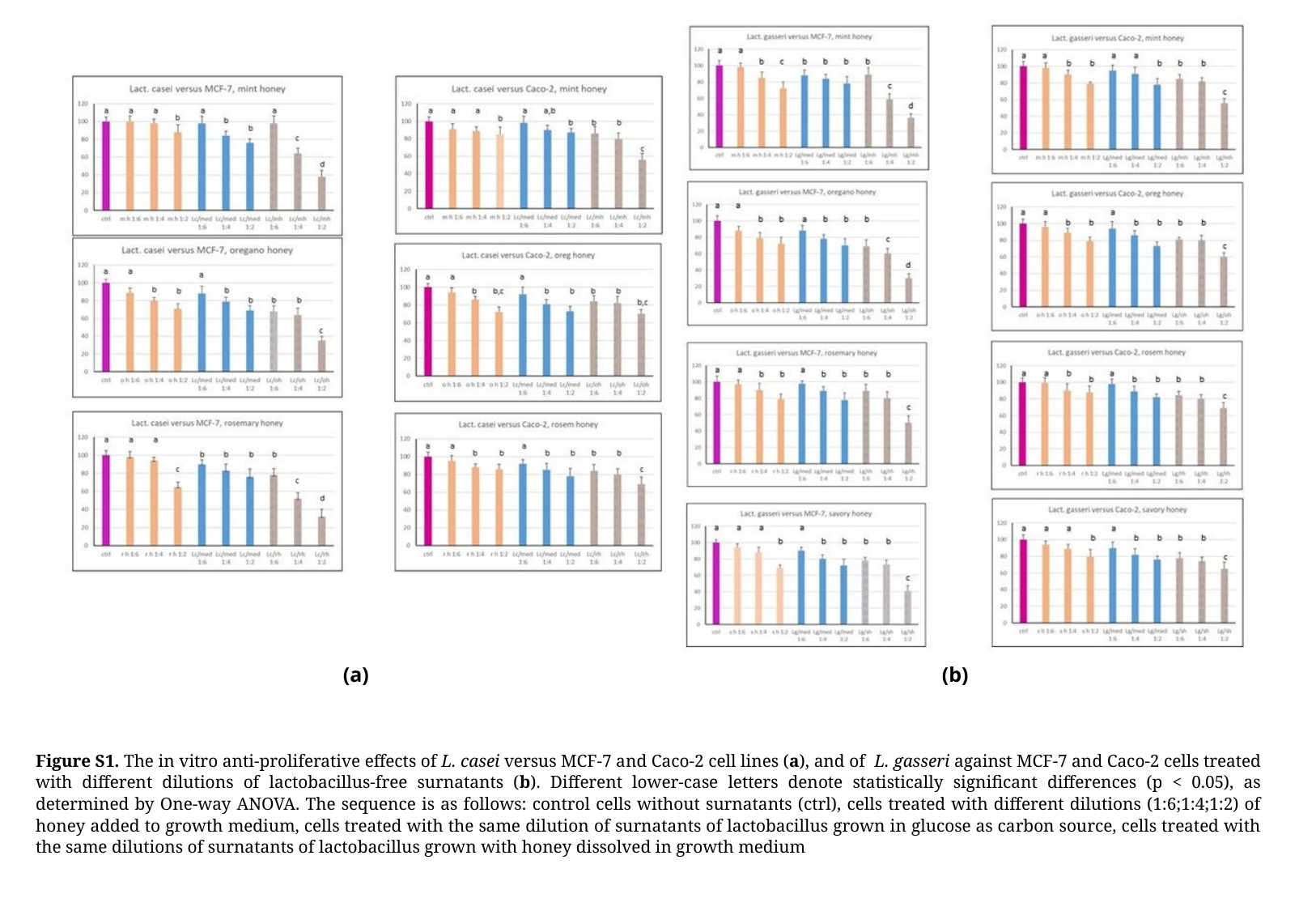

(a)
(b)
Figure S1. The in vitro anti-proliferative effects of L. casei versus MCF-7 and Caco-2 cell lines (a), and of L. gasseri against MCF-7 and Caco-2 cells treated with different dilutions of lactobacillus-free surnatants (b). Different lower-case letters denote statistically significant differences (p < 0.05), as determined by One-way ANOVA. The sequence is as follows: control cells without surnatants (ctrl), cells treated with different dilutions (1:6;1:4;1:2) of honey added to growth medium, cells treated with the same dilution of surnatants of lactobacillus grown in glucose as carbon source, cells treated with the same dilutions of surnatants of lactobacillus grown with honey dissolved in growth medium

## Slide 6
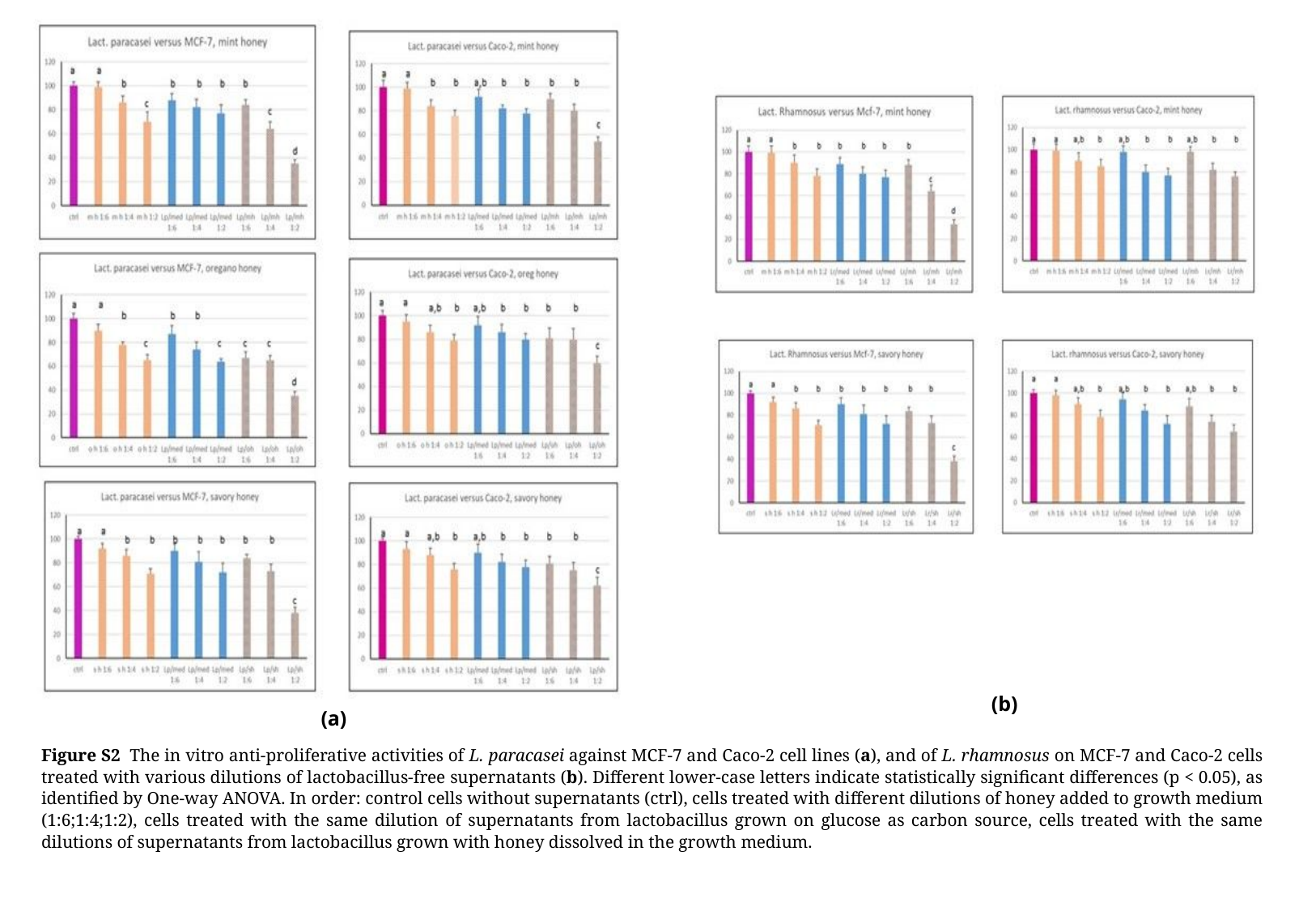

(b)
(a)
Figure S2 The in vitro anti-proliferative activities of L. paracasei against MCF-7 and Caco-2 cell lines (a), and of L. rhamnosus on MCF-7 and Caco-2 cells treated with various dilutions of lactobacillus-free supernatants (b). Different lower-case letters indicate statistically significant differences (p < 0.05), as identified by One-way ANOVA. In order: control cells without supernatants (ctrl), cells treated with different dilutions of honey added to growth medium (1:6;1:4;1:2), cells treated with the same dilution of supernatants from lactobacillus grown on glucose as carbon source, cells treated with the same dilutions of supernatants from lactobacillus grown with honey dissolved in the growth medium.
